# Jianwei Xiaoshi oral liquid attenuates high-calorie diet-induced dyspepsia in immature rats via regulating the pancreatic secretion pathway and maintaining the homeostasis of intestinal microbiota

**DOI:** 10.1186/s13020-024-01052-3

**Published:** 2025-01-04

**Authors:** Yan Zhang, Xiaolu Wei, Shan Jiang, Wenya Gao, Kun Wang, Hongjie Wang, Huijun Wang, Nan Si, Yanyan Zhou, Keke Luo, Mengxiao Wang, Yuyang Liu, Lihua Chen, Liqi Ni, Haiyu Zhao

**Affiliations:** 1 State Key Laboratory for Quality Ensurance and Sustainable Use of Dao-Di Herbs, Institute of Chinese Materia Medica, China Academy of Chinese Medical Sciences, Beijing, 100700 China; 2 Jichuan Pharmaceutical Group Co., Ltd., Jiangsu, 22544 China

**Keywords:** Jianwei Xiaoshi oral liquid, Gut flora, Short-chain fatty acids, Transcriptome, High-calorie diet-induced dyspepsia

## Abstract

**Background:**

Jianwei Xiaoshi oral liquid (JWXS), a classical traditional prescription comprising various edible medicinal plants, has demonstrated significant efficacy in treating paediatric indigestion. It originates from Jianpi Pill, which is developed in the Ming Dynasty and nourishes the spleen and regulates gastrointestinal function. However, the specific molecular mechanisms involved remain unclear.

**Methods:**

To elucidate the material base of JWXS and its underlying mechanism in treating dyspepsia, the UHPLC–Q–Orbitrap HRMS method and network pharmacology were utilized. This was followed by pharmacological experiments, transcriptomics analyses and gut microbiota studies to further investigate the effects of JWXS on dyspepsia.

**Results:**

A total of 105 compounds, mainly flavonoids, alkaloids, organic acids and cyclic peptides, were identified. According to the five principles of generic drug properties, 43 candidate compounds were screened out. Their efficacy was verified through gastric emptying and intestinal propulsion experiments. Transcriptomic analysis revealed that JWXS primarily alleviated dyspepsia symptoms by regulating the secretion of 8 key proteins in the pancreatic secretion pathway. The differences in the gut microbiota, as identified through 16S rRNA and ITS2 sequencing, were subsequently more pronounced than those observed in the bacterial microbiota of the model group. In total, 15 differential bacteria and 16 differential fungi were identified. Targeted metabolomics analysis of SCFAs revealed a significant decrease in valeric acid (VA), acetic acid (AA), and isovaleric acid (IVA) levels in the model group, which were restored to the corresponding levels after the administration of JWXS. Correlation analysis revealed that VA, AA, and IVA were positively correlated with *Lactobacillus* and *Bacteroide*s, and negatively correlated with *Aspergillus* and *Candida*. This further suggested that JWXS might alleviate symptoms of indigestion by regulating the composition of the microbiota, increasing the variety and quantity of beneficial bacteria, reducing fungal contamination, and further increasing the levels of SCFAs in the body.

**Conclusion:**

JWXS improved functional dyspepsia in immature rats via a mechanism involving the regulation of the secretion of 8 key proteins in the pancreatic secretion pathway and the amelioration of flora disorders.

**Graphical Abstract:**

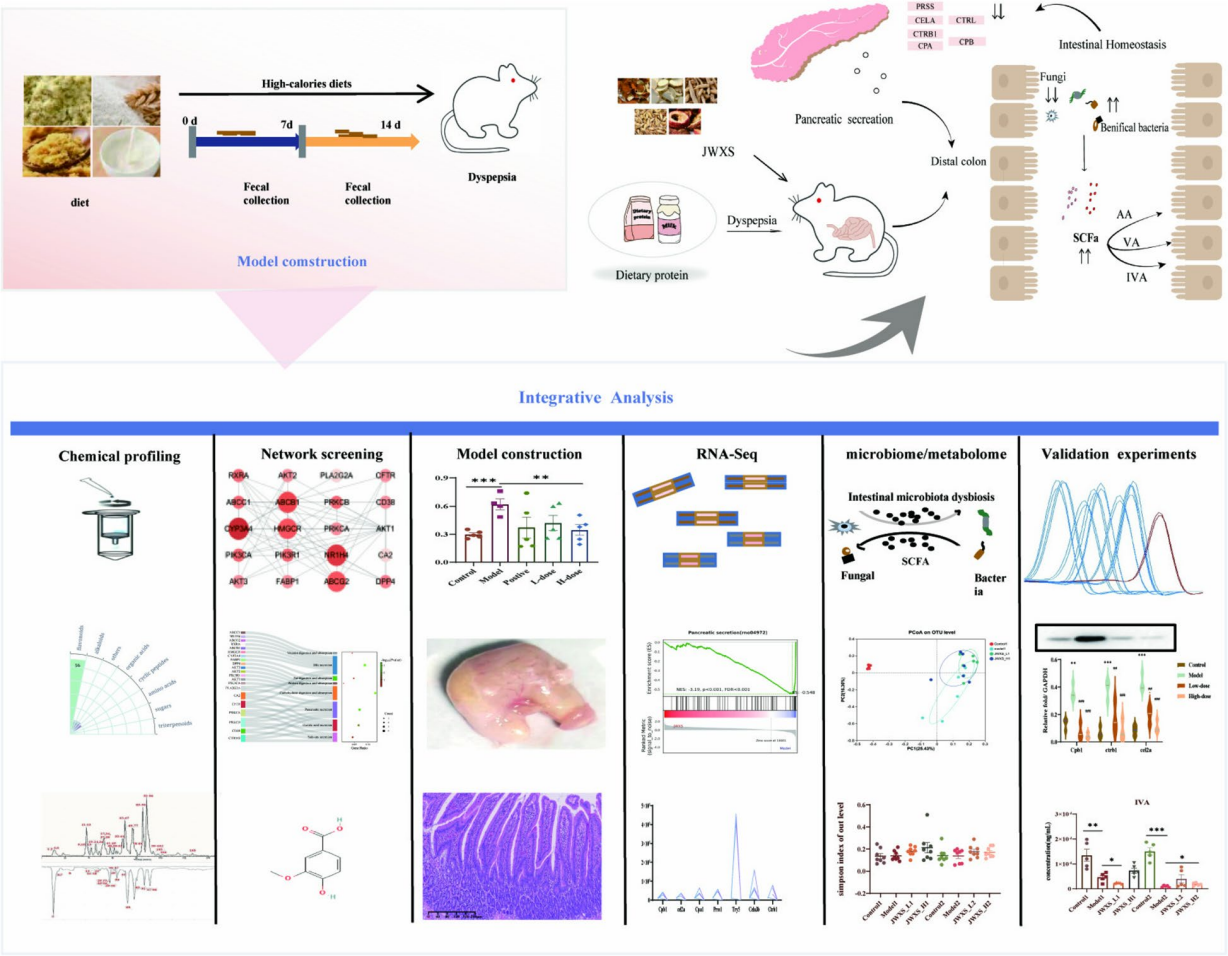

**Supplementary Information:**

The online version contains supplementary material available at 10.1186/s13020-024-01052-3.

## Background

Dyspepsia, commonly referred to as digestive dysfunction, is not a standalone disease but rather a spectrum of symptoms indicative of either functional or organic anomalies within the digestive tract. It manifests in two primary forms: organic indigestion and functional dyspepsia (FD). Factors such as gender, age, race, diet, and environment have been identified as contributing to its pathogenesis [1, 2]. FD is a common chronic nonorganic gastrointestinal disease. To date, the aetiology of FD remains elusive. FD is widely believed to be associated with delayed gastrointestinal emptying, altered gastrointestinal hormone levels, a damaged duodenal barrier and a disordered gut microbiota [3, 4]. The gut microbiota might play a significant role in the pathophysiological processes of FD. Studies have shown that the gut microbiota and the brain‒gut axis maintain a bidirectional connection, thereby regulating gastrointestinal function [5, 6]. Additionally, studies have indicated that modifying the type and composition of the gut microbiota could serve as a safe and efficacious therapeutic approach for alleviating FD symptoms [7, 8]. Moreover, the digestive enzymes secreted by the pancreas significantly influence the composition and function of the gut microbiota, and the metabolites produced by the gut microbiota can, in turn, impact the function and disease status of the pancreas [9]. When pancreatic secretion function is impaired, these nutrients may not be fully digested, resulting in symptoms such as bloating, abdominal pain, flatulence, and steatorrhea. Therefore, evaluating pancreatic secretion function and providing timely treatment for patients with indigestion are crucial [10]. JWXS, a blend of five medicinal and edible homologous herbs, including *Pseudostellariae Radix* (Taizishen), *Citrus Reticulatae Pericarpium* (Chenpi), *Crataegi* Fructus (Shanzha), *Dioscoreae Rhizoma* (Shanyao) and *Hordei Fructus Germinatus* (Maiya), has been used clinically to alleviate dyspepsia, particularly in children and elderly individuals. In clinical practice, it can be utilized either as a standalone treatment or in combination with other medications, such as domperidone or cisapride. Research has demonstrated that JWXS is a traditional Chinese patent medicine, leading to a low incidence of side effects, which can improve the gastric emptying rate and reduce adverse reactions to long-term medication [11]. In the formula, *Pseudostellariae Radix* is a sovereign herb known for its ability to strengthen the spleen and aid in digestion, which is accompanied by the ability of *Citrus Reticulatae Pericarpium* to regulate and strengthen the spleen and enhance digestion to alleviate stagnation. When combined with *Pseudostellariae Radix*, *Dioscoreae Rhizoma*, which nourishes the spleen and stomach, further strengthens the spleen and aids in digestion. Both are classified as ministerial herbs. *Hordei Fructus Germinatus* aids in digestion by invigorating the spleen and enhancing appetite. *Crataegi* Fructus can be used to resolve all kinds of food stagnation, particularly flesh and greasy indigestion. *Hordei Fructus Germinatus* aids in digestion and resolves accumulation. These two compounds serve as adjuvant herbs. Together with the other three herbs, these compounds work synergistically to enhance digestion. The JWXS formula was modified from jianpi pill in the Ming Dynasty (Prescriptions for Universal Relief, Standards for Diagnosis and Treatment, Parenting Family Secrets), Qing Dynasty (Collected Exegesis of Recipes, Cihang set) and Republic of China (Paediatric criterion), which involve invigorating the spleen, conditioning stomach qi and promoting digestion. Notably, in 2020, the share of traditional Chinese patent medicines for stomach strengthening and digestion accounted for a significant portion (47.06%) of the pharmaceutical market in urban public hospitals in China. To date, studies on JWXS have focused mainly on establishing quality standards and observing clinical efficacy [12]. However, investigations into its active components and the mechanism of digestion have been limited.

Consequently, the purpose of this study was to elucidate the material basis of JWXS and uncover its mechanism in ameliorating FD. This study commenced with the identification of the bioactive compounds of JWXS via LC‒MS and network pharmacology. An integrated approach encompassing pharmacological experiments, transcriptomics, gut flora analysis (16S rRNA and ITS2 techniques) and metabolomics was subsequently employed to investigate the efficacy and mechanism of JWXS in immature rats. Additionally, the study examined the implications of these variations on host health and the underlying mechanisms. This research provides a theoretical foundation for the judicious use of JWXS in dyspepsia treatment.

## Materials and methods

### Chemicals and reagents

High-calorie feed preparation: Fish floss was ground into powder and then mixed with soybean powder, flour and milk powder at a ratio of 1:2:1:1. Similar to regular feed, this mixture was shaped into small pieces, baked in an electrothermal constant-temperature blast drying oven at 40 °C to 45 °C, dried, sterilized and stored in a refrigerator at 4 °C for later use.

A custom-made high-protein mixture of milk powder and water was combined at a mass ratio of 52:48 and stirred evenly to make a milk solution with a mass concentration of 520 g/L, which was freshly prepared before each gavage.

High-calorie feed was collected in Beijing Anmosais Biotechnology Co., Ltd. (Beijing, China). The chemical reference substances for component analysis were obtained from Beijing Saibaicao Technology Co., Ltd. (Beijing, China), including quinic acid, citric acid, chlorogenic acid, rutin, quercetin, synephrine, neohesperidin, naringin, hesperetin, nobiletin and ursolic acid. The purity of all standards was greater than 98% by HPLC. The acetic acid (AA), propionic acid (PA), butyric acid (BA), isobutyric acid (IBA), valeric acid (VA), isovaleric acid (IVA), hexanoic acid (HA) and 4-Methylvaleric acid (4-MA, internal standard) were purchased from Sigma-Aldrich (Missouri, USA). JWXS and cisapride were offered by Jichuan Pharmaceutical Group Co., Ltd (Jiangsu, China) and Jingxin pharmaceutical Co., Ltd. (Zhejiang, China), respectively.

AccuBond^II^ SPE ODS-C_18_ cartridges (100 mg, 1 mL) and ProElut C_18_ cartridges (50 mg, 1 mL) were purchased from Beijing Dikma Technology Co., Ltd. (Beijing, China) and Agilent Technologies Co., Ltd. (Beijing, China), respectively.

MS-grade acetonitrile and methanol were obtained from Fisher Scientific (Fair Lawn, NJ, USA); deionized water was produced by a Milli-Q water system (Millipore, Bedford, MA, USA); MS-grade formic acid was purchased from Thermo Fisher Scientific (San Jose, CA, USA); and other chemicals and solvents were of analytical grade.

### Animals and grouping

Seventy-five SPF newborn male Sprague–Dawley (3–4-week-old) rats were procured from Beijing Vital River Laboratory Animal Technology Co., Ltd. and housed under a natural light–dark cycle at a temperature of 25 ± 2 ℃and a relative humidity of 55 ± 10%. The animals were allowed access to food and water ad libitum.

The animals were randomly divided into 5 groups: the control, model, positive (cisapride, 5 mg/kg/d), high-dose (4.28 mL/kg/d) and low-dose groups (2.14 mL/kg/d).

All groups except the control were fed high-calorie feed. From the second day, the 52% milk mixture (2 mL/100 g) was administered bidaily via gavage [13, 14]. Treatment commenced from the first day, with the control group receiving saline. The rats were consistently weighed via the same scale.

## Identification of JWXS components

### Sample preparation

A ProElut C_18_ solid-phase extraction column and AccuBond^II^ SPE ODS-C_18_ cartridge were pretreated for analysis of the JWXS solution. The procedure involved 1 mL of methanol for activation, 1 mL of pure water for equilibration, sample loading, 1 mL of pure water for elution and 1 mL of methanol for elution. The elution mixture was subsequently rotated and evaporated to dryness under N_2_ at 37 ℃ and reconstituted in 100 µL of methanol. After vortexing for 3 min and centrifuging at 12000 rpm at 4 ℃ for 10 min, 10 µL of the supernatant was used for analysis.

### Preparation of standard solutions

Individual standards were dissolved in methanol to prepare single reference substance stock solutions (1.0 mg/mL). These solutions were mixed and diluted with methanol to yield the standard solution (10 µg/mL).

### Instrumentation and experimental conditions

The experiments were performed on a Thermo Fisher Scientific UHPLC system (Dionex UltiMate 3000) coupled with a high-resolution LTQ Orbitrap Velos Pro (USA). The mass spectrometer was equipped with a heated electrospray ionization source. Chromatographic separation was achieved on a Waters ACQUITY UPLC HSS T3 (2.1 × 100 mm, 1.7 µm) at a flow rate of 0.3 mL/min. The injection volume was 10 μL and the column temperature was 35 ℃. The mobile phase A was 0.1% formic acid in water and the mobile phase B was acetonitrile. The elution gradient was optimized as follows: 0–1 min, 0% B; 1–5 min, 0–46% B; 5–11 min, 46–96% B; 11–14 min, 96% B; 14–14.1 min; 96–0% B; 14.1–17 min, 0% B.

LTQ Orbitrap Velos Pro was combined with UHPLC via an ESI interface. Positive and negative ion detection modes were simultaneously applied. The full MS scan range was *m/z* 100–900 and the resolution was 30,000. The acquisition software (Xcalibur 3.0, Thermo) continuously evaluated the full scan survey MS data as it collected and triggered the acquisition of MS/MS spectra depending on preselected criteria. The MS parameters were set as follows: sheath gas flow rate, 40 mL/min; aux gas flow rate, 10 mL/min; capillary temperature, 350 °C; ion spray voltage, ± 3.5 kV; and the MS^2^ experiments were data-dependent scans.

### Network pharmacology analysis

In order to explore the curative components and potential molecular targets of JWXS, network pharmacological analysis was performed. Firstly, compounds were collected based on mass spectrometry identification data with high abundance and supplemented by literature research. The components filtered by GI “absorption” were high, two or more of five indicators "Lipinski, Ghose, Veber, Egan, Muege" were "Yes" and the bioavailability score was greater than or equal to 0.5 (http://www.ch/). Next, the targets of components were predicted on SwissTargetPrediction (http://www.swisstargetprediction.ch/). Then, the disease targets were filtered on TTD (http://db.idrblab.net/ttd/), DrugBank (https://go.drugbank.com/) and GeneCards (https://www.genecards.org/). Only the common targets closely related to JWXS were retained by bioinformatics (http://www.bioinformatics.com.cn/). Subsequently, the hub genes were identified by Cyto-NCA plugin. Finally, the Kyoto Encyclopedia of Genes and Genomes (KEGG) enrichment analysis was performed by OECloud tools (https://cloud.oebiotech.com) and a PPI network was constructed based on hub genes by cytoscape (3.8.2, USA).

### Therapeutic effect of JWXS in high-calorie diets

#### Observation of animals

The variation in daily body weight was monitored to evaluate whether the model was successfully established.

HE pathological staining of the duodenum and analyses of the gastric emptying ratio and percentage of carbon powder intestinal propulsion were performed to evaluate the efficacy of JWXS in immature rats fed a high-calorie diet.

#### Pathological examination

HE staining process: After modelling, the duodenums were separated quickly, rinsed gently with saline, fixed with fresh solution and fixed in 4% paraformaldehyde. Paraffin sections of 4 μm thickness were used for haematoxylin and eosin (H&E) staining. After the samples were fixed well, we strictly followed the SOP procedure for pathological experimental detection to trim, dehydrate, embed, slice, stain, seal and inspect the samples under a microscope.

#### Gastric emptying ratio

The whole stomach from each group was dissected and weighed. The stomach was then split along the small curvature, and the stomach contents were rinsed with physiological saline. After drying with filter paper, the weight of the gastric net was determined, and the percentage of residual gastric content was calculated.

$${\text{Residual rate of gastric content }}\left( \% \right)\, = \,\left[ {\left( {{\text{total weight of stomach}}\,{-}\,{\text{net weight of stomach}}} \right)/{\text{body weight}}} \right]\, \times \,{1}00\%$$

#### Intestinal propulsion rate

On the 14th day, carbon foam was given by gavage after JWXS/cisapride/normal saline was given by intragastric administration for 30 min. Another 30 min later, the rats were dissected. The lengths of the intestinal carbon foam propulsion and small intestine in the different groups were measured and recorded.

$${\text{Intestinal propulsion }}\left( \% \right)\, = \,{\text{advanced length ofsemisolidpaste}}/{\text{total length of the small intestine}}\, \times \,{1}00\% .$$

### Transcriptome analysis and qRT‒PCR validation

#### Transcriptome analysis

The duodenums were ground into powder with liquid nitrogen. Total RNA was extracted from the duodenum with TRIzol (Qiagen, China) according to the manufacturer’s instructions. RNA purity and quantification were determined via a NanoDrop 2000 spectrophotometer (Thermo Scientific, USA), and RNA integrity was assessed via an Agilent 2100 Bioanalyzer (Agilent Technologies, Santa Clara, CA, USA). The transcriptome library was constructed via the VAHTS Universal V5 RNA-seq Library Prep Kit (Vazyme, China) following the manufacturer’s protocol. Transcriptome sequencing and analysis were performed by Shanghai Ouyi Biotechnology Co., Ltd. (Shanghai, China).

Fastp software was used to process raw reads into clean sequences. The clean data were mapped to compare with the GCF_015227675.2_mRatBN7.2 using HISAT2. The FPKM of each gene was calculated using Cufflinks and the sequence counts for each gene were obtained by HTSeqcount. Differential expression analysis was performed using the DESeq (2012) R package. *P* value < 0.05 and fold-change (FC) > 2 or FC < 0.5 were set as the thresholds for differential expressed genes (DEGs). GO (http://www.geneontology.org/) and KEGG pathway enrichment analysis (https://www.genome.jp/kegg/) of DEGs were performed to demonstrate the significantly enriched GO terms and metabolic pathways of DEGs in different groups.

#### **qRT-PCRvalidation**

 To validate the results of the RNA-seq differential gene expression analysis, quantitative real-time PCR (qRT‒PCR) was carried out on specific digestive system components (intestines). *β*-actin was used as an internal reference gene to normalize the gene expression level. The primer sequences were designed in the laboratory and synthesized by TsingKe Biotech on the basis of the mRNA sequences obtained from the NCBI database (Additional file 1: Table S1). qRT‒PCR was conducted using a Roche LightCycler 480 (Roche, Switzerland). Four biological and four technical replicates were used for each gene.

Reverse transcription was carried out via the use of HiScript III RT SuperMix for qPCR (+ gDNA wiper) (Vazyme, Cat. No. R323-01). Each RT reaction consisted of 1 μg of RNA, 4 μL of gDNA remover and 2 μL of 5 × HiScript III qRT SuperMix for qPCR, with a total volume of 20 μL. qRT‒PCR was performed in a 20 μL reaction mixture including 10 μL of Super Mix (2 ×), 1 μL of cDNA (10 μM), 6.36 μL of ddH_2_O and 2.64 μL of primer (10 μM/L). The PCR procedure was as follows: 95 ℃ for 10 s, followed by 40 cycles of 95 ℃ for 5 s and 65 ℃ for 1 min, with a 30 s elapse time for each cycle. The relative expression was estimated via the 2^–ΔΔCt^ method, with control group individuals used as a calibration control [15]. The relative expression results are presented as the fold change relative to that of the control individuals. Statistical significance (*P* < 0.05) was determined via one-way ANOVA.

#### Western blot analysis

Proteins were extracted with RIPA buffer supplemented with protease inhibitor cocktail and separated via SDS‒PAGE, followed by electrotransfer to polyvinylidene fluoride (PVDF) membranes. The samples were subsequently incubated with the corresponding primary antibodies, which included a Ctrb1 antibody (1:1000; Cell Signaling Technology, USA), a Cpa1 antibody (Cell Signaling Technology, USA), a Cel2a antibody (Cell Signaling Technology, USA) and a secondary antibody. The protein bands were visualized using an Enhanced Chemiluminescence Plus detection system (Thermo Fisher, MA, USA) with an enzyme-linked chemiluminescence reagent. The protein concentration was semiquantified via ImageJ (version 1.8.0; National Institutes of Health, Bethesda, MD, USA) and normalized to the corresponding quantification. GAPDH served as a loading control for total protein.

#### 16S rRNA and ITS2 amplicon sequencing

The faecal samples were collected for 0–6 h in metabolic cages after one week and at the end of modelling. The samples were then frozen on dry ice for gut microbiota detection. DNA was extracted via the E.Z.N.A. Rsoil kit (Omega Biotek, Norcross, GA, U.S.) according to the manufacturer’s protocol, detected via 1% agarose gel electrophoresis and quantified via a NanoDrop 2000 UV‒vis spectrophotometer (Thermo Scientific, Wilmington, USA). The primers used for amplification of the 16S V3-V4 region were 338F (5'-TGCTGCCTCCCGTAGGAGT-3') and 806R (5′-GGACTACHVGGGTWTCTAAT-3′). The primers used for ITS2 amplification were 5'-CTTGGTCATTTAGAGGAAGTAA-3' and 5'-GCTGCGTTCTTCATCGATGC-3', which were modified with a sequencing universal connector and a sample-specific barcode sequence. The PCR amplification products were verified via 2% agarose gel electrophoresis, purified via an AxyPrep PCR Cleanup Kit (Axygen, USA) and quantified via a QuantiFluor™-ST Blue fluorescence quantitative system (Promega, China). The amplification products were sequenced at 2 × 300 bp paired ends via the MiSeq Reagent Kit V39t (600 cycles) on an Illumina MiSeq PE300 platform by Chengdu Lilai Biotechnology Co., Ltd. (Chengdu, China). The sequences were spliced via FLASH software (FLASH v1.2.1.1) (Magoc & Salzberg, 2011) and filtered via Vsearch software (Vsearch v2.3.4). Taxonomy assignment was performed against the SILVA database (v138) in the case of bacteria, whereas the UNITE (v8.0) database was used for fungi. Rarefaction curves were computed for all the samples, and the relative abundance was subsequently estimated.

#### Short-chain fatty acids analyse

Fecal samples were ground into small graininess mixture. 30 mg fecal and 500 μL acetonitrile (containing 0.5% concentrated hydrochloric acid) were placed into a 1.5 mL centrifuge tube to homogenize at 3000 rpm/min for 180 s. The homogenates were centrifuged (12,000 r/min, 4 ℃) for 15 min and the supernatant was transferred to a new EP tube for later assay. The supernatant 190 μL and internal standard (100 μg/mL) 10 μL were mixed for testing. Samples were analyzed by 7000C GC-TQ gas chromatography–mass spectrometry (Agilent Technologies, Santa Clara, CA, USA).

The chromatographic column was a DB-FFAP (30 m × 0.25 mm i.d., 0.25 µm film, Agilent Technologies). Helium was used as a carrier gas at a constant flow rate of 1 mL/min. The oven program was set to the initial temperature of 50 ℃ for 2 min and then increased to 120 ℃ at a rate of 15 ℃/min, 170 ℃ at a rate of 5 ℃/min, 240 ℃ at a rate of 15 ℃/min and held at 240 ℃ for 3 min finally. The data were acquired in full scan mode and SIM mode (EI at 70 eV, ion source temperature at 250 ℃). The parent ion of AA, PA, BA, IBA, VA, IVA and 4-MA were *m/z* 43, 74, 43, 60, 60, 60, 60, and 55, respectively.

#### Data statistical analysis

All quantitative data were expressed as the mean ± standard error of the mean (SEM) and were analysed via unpaired Student’s t test for two groups and one-way ANOVA for multiple groups. Statistical calculations were performed with statistical significance, denoted by *p* ≤ 0.05. Spearman correlation analysis was conducted among genes, bacteria and fungal flora. Histograms were drawn using GraphPad Prism 8.0 (GraphPad Software, USA).

## Results

### Chemical composition analysis of JWXS

**Fig. 1:**
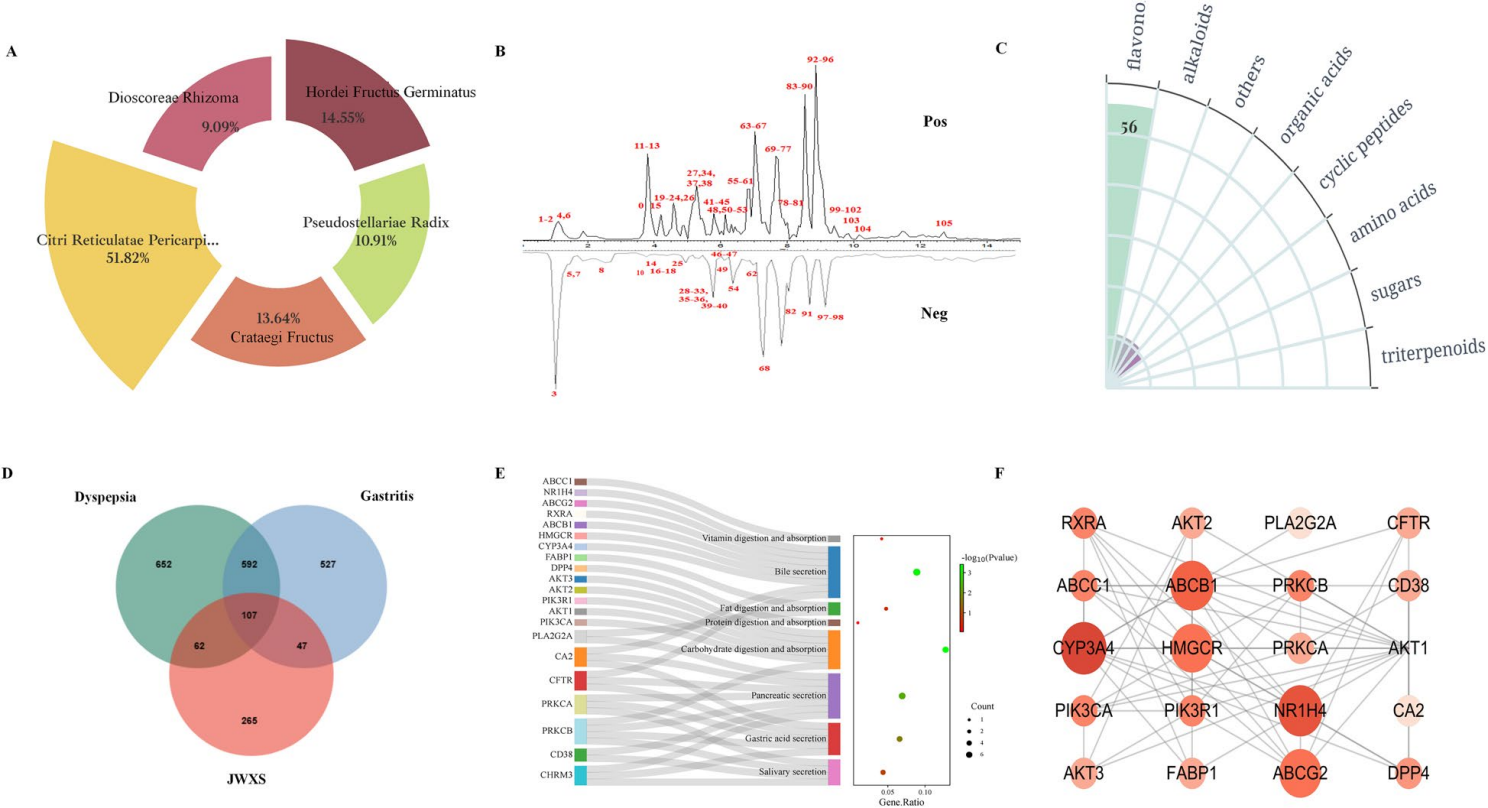
Identification compounds and genes in JWXS against dyspepsia. **A** The sources of each compounds. **B** The base peak chromatogram JWXS. **C** The classes of compounds. **D** Intersection analysis among the diseases genes of gastritis and dyspepsia and putative JWXS target genes. **E** KEGG pathways enriched in the gastrointestinal and digestive systems. **F** A PPI network with the 21 genes. The color depth and node size were determined by the degree value

A total of 105 compounds were identified by comparing the precise relative molecular weights, ion fragments, reference materials and literature sources [16–22]. Their mass profile information and chromatographic peaks were detailed in Table 1 and Fig. 1A, B. The main chemical compounds included 56 flavonoids, 11 alkaloids, 1 triterpenoid, 11 organic acids, 10 cyclic peptides, 3 amino acids, 2 sugars and 11 compounds from other classes (Fig. 1C). Notably, 11 compounds, namely, synephrine, citric acid, chlorogenic acid, quinic acid, rutin, quercetin, neohesperidin, naringin, hesperetin, nobiletin and ursolic acid, were unambiguously determined by comparison with standard substances.

**Table 1:** Chemical constituents identification of JWXS in positive and negative ion mode

No	Rt (min)	Ion Mode	Molecular Formula	Theoretical value ( *m/z)*	Caculated value ( *m/z*)	Error (ppm)	Characteristic ion peak of MS^n^	Identifification Compound	Source
1	1.01	+	C_11_H_14_N_2_	175.1229	175.1195	1.90	60.0556,70.0652,116.0711	Gramine	A
2	1.05	+	C_6_H_14_N_4_O_2_	175.1190	175.1182	−4.07	112.08636,116.07010,130.09695,158.09181	Arginine	C, D
3	1.08	−	C_6_H_12_O_6_	179.0550	179.0559	5.17	131.0352,143.0351,161.0453	D-Sucrose	B
4	1.12^F^	+	C_9_H_13_NO_2_	168.1019	168.1020	0.39	91.0541,107.0491,135.0680	Synephrine	C
5	1.13^F^	−	C_7_H_12_O_6_	191.0550	191.0557	3.80	85.0295,127.0401,173.0453	Quinic acid	D
6	1.27	+	C_12_H_22_O_11_	343.1235	343.1219	− 4.66	145.04869,163.05914	Isomaltose	A
7	1.48	−	C_6_H_8_O_7_	191.0186	191.0194	3.93	111.0089	Isocitric acid	D
8	2.55^F^	−	C_6_H_8_O_7_	191.0186	191.0193	3.62	111.0089,173.0092	Citric acid	D
9	3.55	+	C_6_H_13_NO_2_	132.1019	132.1021	1.55	86.0965,115.0757	L-Leucine	B
10	3.79	−	C_19_H_24_O_4_	315.1591	315.1590	2.76	147.0816,163.0763,297.1492	1,7-Bis(4-hydroxyphenyl)−3,5-heptanediol	C
11	3.8	+	C_10_H_13_N_5_O_4_	268.1040	268.1042	0.71	136.0624,152.0584	Adenosine	A
12	3.92	+	C_9_H_13_NO	152.1069	152.1072	1.44	103.0542,121.0652	N-methyltyramine	A
13	3.97	+	C_10_H_15_NO	166.1226	166.1229	1.80	91.0542,103.0543,121.0650	Hordenine	A
14	4.17	−	C_6_H_6_O_3_	127.0390	127.0392	1.73	99.0440,109.0287	Phloroglucinol	D
15	4.20	+	C_9_H_11_NO_2_	166.0863	166.0865	1.23	103.0545,120.0814	Phenylalanine	A,B
16	4.37	−	C_9_H_17_NO_5_	218.1023	218.1026	1.24	88.0403,146.0820	D-Pantothenic Acid	B
17	4.45	−	C_8_H_8_O_4_	167.0339	167.0347	5.12	93.0346,123.0460	Vanillic acid	B
18	4.56^F^	−	C_16_H_18_O_9_	353.0867	353.0865	− 0.62	135.0453,179.0452,191.0564	Chlorogenic acid	D
19	4.58	+	C_14_H_20_N_4_O_3_	291.1463	291.1452	0.22	85.0295,119.0503	Coumaroyl-hydroxyagmatine	A
20	4.61	+	C_16_H_24_N_4_O_5_	353.1819	353.1829	2.67	336.1670	Sinapoyl-hydroxyagmatine	A
21	4.74	+	C_15_H_22_N_4_O_4_	323.1714	323.1718	1.42	145.0289,177.0555,305.1625	Feruloyl-hydroxyagmatine	A
22	4.75	+	C_14_H_20_N_2_O_3_	265.1547	265.1551	1.59	177.0553,248.1293	Feruloyl putrescine	A
23	4.79	+	C_27_H_30_O_16_	611.1607	611.1612	0.87		Lucenin-2	C
24	4.87	+	C_14_H_20_N_4_O_2_	277.1659	277.1653	1.29	147.0443,217.1337	p-Coumaroylagmatine	A
25	4.89	−	C_16_H_18_O_9_	353.0867	353.0868	0.23	135.0450,191.0556	4-Dicaffeoylquinic Acid	D
26	4.95	+	C_27_H_30_O_15_	595.1657	595.1664	1.06	457.1158,529.1376,577.1587	Kaempferol-3-O-glucose-rhamnoside	D
27	5.01	+	C_15_H_22_N_4_O_3_	307.1765	307.1769	1.44	177.0562,290.1539	Sinapoyl agmatine	A
28	5.03	−	C_27_H_30_O_15_	593.1501	593.1491	− 1.66	325.0728,353.0682,383.0791,473.1111,503.1212	Vicenin-2	C
29	5.03	−	C_27_H_30_O_15_	593.1501	593.1491	−1.66	285.0401,353.0682,383.0791,473.1111,503.1212	Lonicerin	C
30	5.03	−	C_27_H_30_O_15_	593.1512	593.1481	− 3.32	353.0682383.0791,473.1111	Saponarin	A
31	5.03	−	C_11_H_12_O_6_	239.0550	239.0557	2.91	149.0608,177.0557,179.0348	Eucomic acid	B
32	5.23	−	C_26_H_28_O_14_	563.0395	563.1395	0.00	353.0681,383.0791,473.1109,503.1210	Vitexin-O-xyloside/Apiin	C,D
33	5.24	−	C_9_H_8_O_4_	179.0338	179.0347	4.61	135.0452	Caffeic acid	D
34	5.44^F^	+	C_27_H_30_O_16_	611.1607	611.1592	− 2.42		Rutin	C,D
35	5.44	−	C_27_H_32_O_15_	595.1657	595.1650	− 1.29	287.0573,433.1509,549.2939	Eriocitrin/Neoeriocitrin	C
36	5.52	−	C_27_H_34_O_15_	597.1814	597.1818	0.61		Phloretin-3',5'-di-C-glucoside	C
37	5.55	+	C_26_H_30_O_8_	471.2013	471.2021	1.58		Limonin isomer	C
38	5.61^F^	+	C_15_H_10_O_7_	303.0499	303.0544	1.62	275.0565,285.1253	Quercetin	B,D,E
39	5.67	−	C_21_H_18_O_12_	463.0871	463.0886	− 1.06	151.0039,287.0562,301.0357	Hyperoside	D
40	5.69	−	C_21_H_20_O_11_	447.0922	447.0897	− 5.52		Orientin	C
41	5.71	+	C_22_H_22_O_11_	463.1235	463.1241	1.21	257.0929,343.0826,367.0826,445.1144	Diosmetin /Chrysoeriol-C-glucoside	C
42	5.83	+	C_28_H_32_O_16_	625.1763	625.1768	0.81	317.0675,463.1257, 479.1214	Diosmetin-6,8-di-*C*-glucoside[chrysoeriol-6,8-di-*C*-glucoside]	C
43	5.89	+	C_28_H_34_O_15_	609.1814	609.1793	− 3.51	286.0493,301.0724,463.1260	Hesperidin	C
44	5.96	+	C_29_H_32_O_17_	653.1712	653.1717	0.71	332.0544,347.0780,455.0992	Limocitrin-3-OHMG-*β*-glucoside	C
45	5.97	+	C_22_H_22_O_11_	463.1235	463.1236	0.31	255.2122,273.2227,319.2286,427.2502	Diosmetin /Chrysoeriol-C-glucoside	C
46	6.02	−	C_34_H_48_O_16_	711.2859	711.2849	− 1.34		Nomilinic acid Glucoside	C
47	6.09	−	C_34_H_46_O_15_	693.2752	639.2739	− 1.97		Nomilin glucoside	C
48	6.29^F^	+	C_28_H_34_O_15_	609.1814	609.1785	− 4.71	301.0727	Neohesperidin	C
49	6.33	−	C_20_H_24_O_9_	407.1336	407.1333	− 0.81	245.0823,325.2018	Anisodamine saponin A	D
50	6.43	+	C_25_H_35_O_6_N_5_	502.2660	502.2662	0.46	233.1295,261.1246278.1513,361.1890,389.1840,457.2466,474.2731,484.2573	Pseudostellarin A	E
51	6.43	+	C_28_H_34_O_14_	595.2021	595.2025	0.64	287.0929,397.1301,433.1514,449.1468,541.1730	Poncirin	C
52	6.48	+	C_32_H_38_O_17_	695.2182	695.2188	0.83	359.0758,389.1260	Pentamethoxyflavone-3-O-HMG-β-glucoside	C
53	6.49	+	C_32_H_38_O_18_	711.2131	711.2134	0.49	375.0731,377.0886,405.1203	Monohydroxy-pentamethoxyflavonol-3-O-HMG-β-glucoside	C
54	6.55	−	C_19_H_24_O_4_	315.1591	315.1597	1.95	121.0659,149.0609	Hannokinol	B
55	6.60	+	C_31_H_36_O_18_	697.1974	697.1982	1.06	343.0467,361.0577,376.0809,391.1050	7, 4-dihydroxy-5, 6, 8, 3-tetrame-thoxyflavonol-3-O-HMG-β-glucoside	C
56	6.66	+	C_33_H_46_N_8_O_8_	683.3511	683.3517	0.90	342.1829,513.2473,626.3335,665.3439	Pseudostellarin B	E
57	6.67	+	C_36_H_53_N_7_O_9_	728.3978	728.3983	0.77	502.2325,615.3173,700.4075,710.3904	Citrusin III	C
58	6.81	+	C_27_H_32_O_14_	581.1865	581.1874	1.65	419.1366	Narirutin	C
59	6.86	+	C_33_H_40_O_18_	725.2287	725.2298	1.46	223.0607,389.0894,419.1366	Naringin-3-*O*—(3-hydroxy-3-methylglutarate)—glucoside	C
60	6.93	+	C_32_H_38_O_18_	711.2131	711.2141	1.35	375.0736,377.0885,405.1200	monohydroxy-pentamethoxyflavonol-3-O-HMG-β-glucoside	C
61	7.01	+	C_40_H_58_N_8_O_8_	779.4450	779.4462	1.53	377.2575,405.2525,552.3218,751.4553	Heterophyllin B	E
62	7.09	−	C_18_H_32_O_5_	327.2177	327.2175	2.66	171.1031,183.1394,211.1342,229.1449	9,10-dihydroxy-8-oxo-12-octadEcenoic acid	A
63	7.11^F^	+	C_27_H_32_O_14_	581.1865	581.1874	1.55	419.1363	Naringin	C
64	7.16	+	C_34_H_53_N_7_O_9_	704.3978	704.3987	1.32	514.3052,591.3180,668.3813,686.3915	Citrusin I	C
65	7.21	+	C_38_H_56_O_10_N_8_	785.4192	785.4196	0.44	308.1984,438.3099,585.3816,629.3329,668.3436,767.4127	Pseudostellarin F	E
66	7.23	+	C_40_H_60_N_8_O_10_	813.4505	813.4506	0.13	471.2974,629.3693,666.3212,795.4439	Pseudostellarin C	E
67	7.29	+	C_36_H_55_N_7_O_8_	714.4185	714.4194	1.23	211.1451,324.2229	Pseudostellarin D	E
68	7.32	−	C_18_H_34_O_5_	329.2333	329.2331	2.64	211.1347,311.2233	Pinellic Acid	A
69	7.41^F^	+	C_16_H_14_O_6_	301.0706	301.0714	2.38	164.0114,199.0401,242.0582,286.0479	Hesperetin	C
70	7.49	+	C_20_H_20_O_8_	389.1231	389.1237	1.45	356.0911,361.0938,374.1017	Hydroxy-pentamethoxy flavonoid/Hydroxy-pentamethoxy flavonoid isomer	C
71	7.53	+	C_37_H_57_N_7_O_8_	728.4341	728.4356	2.02	197.1300,310.2133,457.2837,514.3055,627.3903,710.4286	Heterophyllin A	E
72	7.60	+	C_20_H_20_O_7_	373.1282	373.1287	1.37	315.0867,343.0837,358.1071	Isosinensetin	C
73	7.65	+	C_42_H_56_N_8_O_9_	817.4243	817.4251	0.99	354.1464,419.1450,467.2302,781.4066,799.4177	Pseudostellarin G	E
74	7.73	+	C_20_H_20_O_8_	389.1231	389.1231	0.04	328.0954,359.0779,374.1013	Hydroxy-pentamethoxy flavonoid/Hydroxy-pentamethoxy flavonoid isomer	C
75	7.77	+	C_45_H_67_N_9_O_9_	878.5135	878.5129	− 0.65	301.2942,849.4865	Pseudostellarin E	E
76	7.90	+	C_21_H_22_O_8_	403.1392	403.1387	1.18	165.0554,342.1114,373.0936,388.1173	5,6,7,3,4',5'-hexamethoxyflavone	C
77	8.00	+	C_20_H_22_O_7_	375.1438	375.1439	0.16	357.1335	5-hydroxy-2- (3-hydroxy-4,5-dimethoxyphenyl) −7,8-dimethoxy-4H-1-benzopyran-4-one	C
78	8.00	+	C_19_H_18_O_6_	343.1176	343.1183	1.88	181.0137,313.0723,328.0955	Tetramethoxyflavone	C
79	8	+	C_19_H_18_O_6_	343.1176	343.1180	1.24	299.0927,313.0723,328.0955	5, 6, 7, 4'-tetramethoxyflavone	C
80	8.08	+	C_20_H_20_O_7_	373.1282	373.1287	1.37	312.1008,343.0830,358.1063	Isosinensetin isomer	C
81	8.10	+	C_20_H_20_O_8_	389.1231	389.1236	1.38	345.0986,359.0778,374.1014	Hydroxy-pentamethoxy flavonoid/Hydroxy-pentamethoxy flavonoid isomer	C
82	8.10	−	C_15_H_16_O_3_	243.1015	243.1021	2.30	122.0373,137.0608	batatasin III	B
83	8.20	+	C_35_H_48_N_6_O_7_	665.3657	665.3665	1.11	330.1828,387.2048,534.2740,548.2899,647.3588	Heterophyllin D	E
84	8.20	+	C_26_H_30_O_8_	471.2013	471.2020	1.33		Limonin	C
85	8.25	+	C_20_H_22_O_7_	375.1438	375.1443	1.39	357.1335	5-hydroxy-2- (3-hydroxy-4,5-dimethoxyphenyl) −7,8-dimethoxy-4H-1-benzopyran-4-one	C
86	8.38	+	C_21_H_22_O_8_	403.1394	403.1387	1.55	165.0555,327.0880,373.0936,388.1171	5,7,8,3,4',5'-hexamethoxyflavone	C
87	8.39	+	C_18_H_16_O_5_	313.1070	313.1075	1.37	269.0821,285.0766,298.0844	5,7, 4'-trimethoxyflavone	C
88	8.44	+	C_20_H_20_O_7_	373.1282	373.1287	1.45	312.1008,343.0831,358.1063	Sinensetin	C
89	8.58^F^	+	C_21_H_22_O_8_	403.1393	403.1387	1.26	342.1115,355.0831,373.0938,388.1173	Nobiletin	C
90	8.58	+	C_19_H_18_O_6_	343.1176	343.1182	1.68	282.0903,299.0930,328.0955	Tetramethoxyflavone isomer	C
91	8.61	−	C_15_H_12_O_4_	255.0652	255.0654	0.72	137.0244, 268.0377	Liquiritigenin	B
92	8.86	+	C_22_H_24_O_9_	433.1493	433.1495	0.53	403.1047,418.1281	3,5,6,7,8,3',4-heptemethoxyfla-vone	C
93	8.89	+	C_22_H_24_O_9_	433.1493	433.1495	0.53	385.0938,403.1045,418.1279	Heptamethoxyflavonoid	C
94	9.03	+	C_19_H_18_O_7_	359.1125	359.1132	1.84	312.1013,326.0804,344.0883	monohydroxy-tet-ramethoxyflavone	C
95	9.03	+	C_20_H_20_O_7_	373.1282	373.1287	1.45	312.1017,343.0840,358.1074	Tangeretin-pentamethoxyflavone	C
96	9.10	+	C_21_H_22_O_9_	419.1337	419.1342	1.20	213.2284,279.2356,343.0834	Gardenin A isomer	C
97	9.15	−	C_30_H_48_O_5_	487.3418	487.3405	− 2.71		2,19-dihydroxyursolic acid	D
98	9.22	−	C_18_H_34_O_4_	313.2384	313.2369	− 1.26	277.2169,295.2274	9,10-dihydroxy-12Z-octadecenoic acid	A
99	9.29	+	C_19_H_18_O_7_	359.1125	359.1132	1.84	301.0728,330.0756,344.0911	Hydroxy-tetramethoxyflavone/Hydroxytetramethoxyflavone isomer	C
100	9.29	+	C_20_H_20_O_8_	389.1231	389.1241	− 0.83	253.1086,265.1085,361.0938,371.1510	Hydroxy-pentamethoxy flavonoid/Hydroxy-pentamethoxy flavonoid isomer	C
101	9.42	+	C_21_H_22_O_8_	403.1394	403.1387	1.55	355.0828,373.0936,388.1169	Nobiletin isomer	C
102	9.49	+	C_20_H_20_O_8_	389.1231	389.1241	2.61	132.1024,283.2643,359.0777,374.1013	Hydroxy-pentamethoxy flavonoid/Hydroxy-pentamethoxy flavonoid isomer	C
103	9.78	+	C_21_H_22_O_9_	419.1337	419.1346	2.13	371.0785,389.0890,404.1122	Gardenin A	C
104	10.00	+	C_19_H_18_O_7_	359.1125	359.1133	2.09	301.0720,329.0672,344.0908	Hydroxy-tetramethoxyflavone/Hydroxytetramethoxyflavone isomer	C
105	12.8^F^	−	C_30_H_48_O_3_	455.3519	455.3502	− 3.98		Ursolic acid	D

^A^*Hordei Fructus* Germinatus.; ^B^*Dioscoreae Rhizoma*; ^C^*Citrus Reticulatae Pericarpium;* ^D^*Crataegi* Fructus; ^E^*Pseudostellariae Radix*

^F^identified by comparison with reference standards

### Network pharmacology analysis

While traditional Chinese medicine (TCM) herbal formulae provide valuable therapeutic strategies, the specifics of their active ingredients and interaction mechanisms remain unclear. In recent years, network pharmacology has emerged as a comprehensive approach to identify curative compounds and elucidate the underlying mechanism of action. By integrating component identification results with literature reviews, 43 compounds were identified through conditional screening, as detailed in Table S2 [23–25]. Combined with the SwissTargetPrediction database, a total of 3433 targets were initially retrieved with a probability threshold of ≥ 0.1; this number was narrowed down to 481 compound targets with numerous commonalities.

Given that dietary stagnation is a prevalent clinical issue, such as in anorexia and gastritis. We used "gastritis" and "dyspepsia" as the keywords in GeneCards and other databases to obtain 1273 and 1413 targets, respectively. A Venn diagram (Fig. 1D) revealed 107 intersecting targets, with 169 related to dyspepsia and 154 related to gastritis. The most direct impact of dyspepsia was on the digestive system. The gastrointestinal and digestive system pathways were distinguished via Kyoto Encyclopedia of Genes and Genomes (KEGG) functional analysis (Fig. 1E). The results are consistent with reality. The duodenum is the primary location for food accumulation and connects the stomach and the small intestine. It plays a crucial role in the collection of various digestive juices, including pancreatic juice, bile, and enzymes; digestion truly begins in the small intestine. Then, 21 hub genes were identified to construct a PPI protein interaction network in line with the degree value (Fig. 1F). The target genes were strongly associated with "gastric acid secretion", "pancreatic secretion", "bile secretion" and "carbohydrate digestion and absorption".

### Polypharmacology verification

**Fig. 2:**
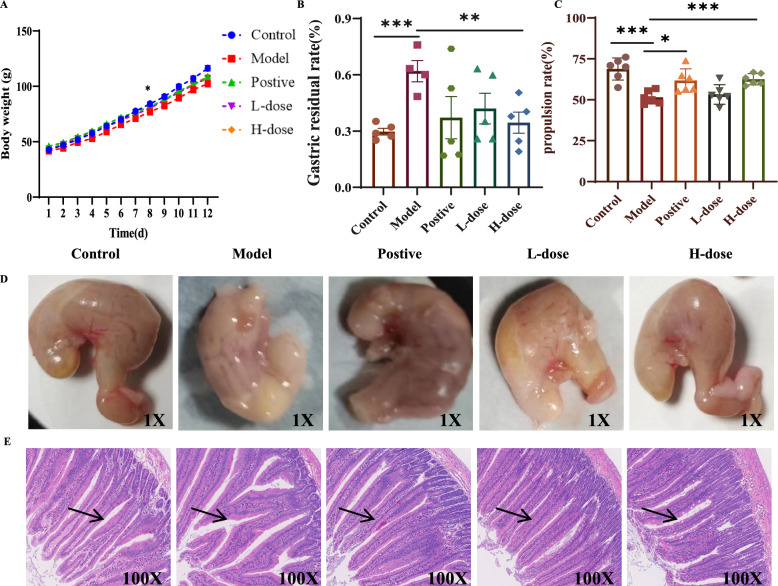
Evaluation analysis of JWXS's efficacy. **A** Daily body weigh records (n = 15). **B** Gastric residual rate (%) (n = 6). **C** propulsion rate (%) (n = 6). **D** Gastric appearance (n = 6). **E** HE staining analysis (n = 6)

JWXS has been used traditionally in China, particularly for children. Thus, the experiments were performed on neonatal rats. As shown in Fig. 2A, there were no initial weight differences between the control and model groups, but beginning on the 6th day, the model group presented symptoms of lethargy, bloating and gastrointestinal disturbances with significant weight gain reduction after 8 days, indicating that the model was established successfully. After JWXS administration, the weight loss of the rats in the JWXS-treated groups was slower than that in the model group.

A high-calorie diet leads to impaired gastrointestinal function, as indicated by gastric emptying function and intestinal propulsion ability. Compared with the model group, JWXS treatment effectively alleviated these symptoms, with high doses showing improvements compared with the positive control drug, cisapride (Fig. 2B, C). Additionally, the model group exhibited gastric surface protrusions, suggesting stomach inflammation, which was alleviated after JWXS treatment (Fig. 2D). The cisapride group still exhibited some inflammation protrusions, indicating its primary role in enhancing intestinal peristalsis.

In this study, congestion, stasis, bleeding, oedema, degeneration, necrosis, proliferation, fibrosis, organization, granulation tissue and inflammatory infiltration were observed via histopathological examination. As shown in Fig. 2E, no significant organic lesions were traced among the groups, which was consistent with the literature [4]. Compared with those in the control group, the villi in the duodenal tissue of the model group were shorter and wider. However, these pathological changes were reversed to varying degrees after treatment with JWXS. The corresponding changes in the HE-stained images has been labeled with black arrows.

### Transcriptomic and qRT‒PCR experiments

Gene expression was first examined in all the rats. Overall, 80.89 giga base (GB) clean data were obtained, with each sample ranging from 6.47 to 7.01 GB and a Q30 score of 92.04% ~ 93.47%. The GC content was distributed in the range of 49.92% ~ 51.28%. A correlation heatmap was generated to show that the data distribution of the gene expression levels in the samples was basically consistent (Fig. S1A). The genome alignment of each sample was subsequently obtained, with an alignment rate of 97.66% ~ 98.34%. The above results showed that the data could be used for further analysis.

**Fig. 3:**
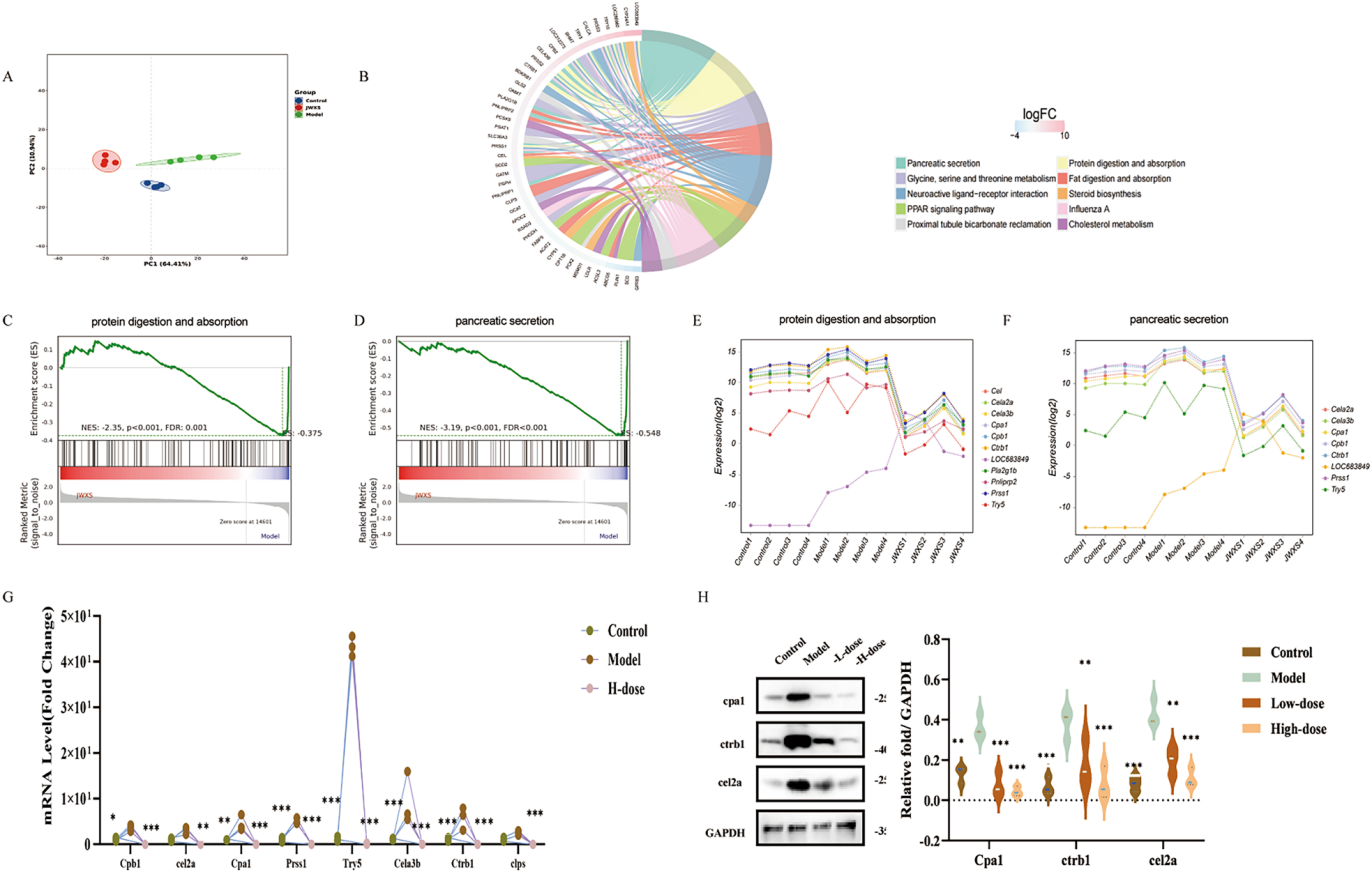
Transcriptome data analysis and validation. **A** PCA diagrams among the control, model and JWXS groups. **B** KEGG pathways significantly enriched with the 105 differential genes. **C** GSEA plot for protein digestion and absorption pathway. **D** GSEA plot for pancreatic secretion pathway. **E** Expression tendency of DEGs of protein digestion and absorption pathway. **F** Expression tendency of DEGs of pancreatic secretion pathway. **G** 8 validated genes by qRT-PCR. **H** WB experiments analysis. GraphPad Prism 8.0 was used for statistical analyses, *P < 0.05, **P < 0.01, ***P < 0.001

Principal component analysis (PCA) effectively distinguished the JWXS, control, and model groups, as depicted in Fig. 3A. The quantification of gene expression in each sample was standardized via DESeq2 [26]. This standardization employs BaseMean values to estimate expression levels, calculate fold changes and conduct significance testing via a negative binomial distribution test (NB). Differential gene expression was identified on the basis of the criteria of a fold change (FC) greater than 2 and a *p* value less than 0.05. Figure S1B illustrated the number of genes uniquely and commonly expressed across different comparative groups. In the model group relative to the control group, a total of 243 DEGs were identified, including 161 upregulated and 82 downregulated genes. The comparison of the JWXS group and the model group revealed 283 DEGs, with 266 upregulated and 17 downregulated genes. Overall, 105 genes were differentially expressed.

Enrichment analysis was performed to categorize functional pathways among 105 DEGs with *p* values < 0.05. As shown in Fig. S1B, the GO enrichment results revealed significant gene clustering in biological processes related to digestion (*p* = 6.97E−10), response to peptide hormones during processing (*p* = 4.76E−07), response to food processing (*p* = 9.86E−04), etc. Additional insights from the cellular component and molecular function GO analyses were presented in Fig. S1C. In addition, the KEGG enrichment demonstrated a predominant concentration of these genes in pathways associated with the digestive system, including pancreatic secretion (*p* = 3.00E−20), protein digestion and absorption (*p* = 3.98E−14) and neuroactive ligand‒receptor interaction (*p* = 2.29E−7), as shown in Fig. 3B. Additionally, the GSEA results suggested that the protein digestion and absorption and pancreatic secretion pathways whose genes were affected by JWXS on a large scale were potentially enriched in the model group and were reversed after JWXS treatment (Fig. 3C, D). The expression tendencies of DEGs related to protein digestion and absorption and the pancreatic secretion pathway were displayed in Fig. 3E, F.

To further validate key regulatory genes and minimize false-positive results, twenty-eight key differentially expressed genes with FPKMs ≥ 2 were identified. Coincidentally, 8 of these DEGs were significantly enriched in the pancreatic secretion pathway and the protein digestion and absorption pathway. Subsequently, eight genes involved in digestive processes, namely, trypsin-5 (try5), chymotrypsin B1 (ctrb1), chymotrypsin-like elastase family member 2A (cel2a), chymotrypsin-like elastase family member 3B (cel3b), carboxypeptidase A1 (cpa1), carboxypeptidase B1 (cpb1), colipase (clps) and serine protease 1 (prss1), were selected for verification by qRT‒PCR analysis. Compared with those in the control group, the contents of the 8 DEGs in the model group were significantly increased, whereas they were only expressed at low levels in the JWXS group (Fig. 3G). This phenomenon suggested that model rats might require increased absorption to counteract the effects of a high-protein diet [27]. Furthermore, three representative proteins (ctrb1, cel2a, and cpa1) were selected for additional validation (Fig. 3H). Compared with those of the control, the protein levels of these genes markedly increased in the model group. Postadministration, a certain degree of normalization of protein expression was observed.

### Composition of bacterial and fungal communities

The microflora is an important factor that causes dyspepsia. To consistently assess the impact of a high-calorie diet in detail, faecal samples were collected for sequencing at two key stages: after one week and at the conclusion of the dietary modelling period. The samples were categorized into distinct groups for analysis, which were named as follows: control1, model1, JWXS_L1 (low dose of JWXS), and JWXS_H1 (high dose of JWXS), followed by control2, model2, JWXS_L2, and JWXS_H2 in the subsequent stage.

### Variation in the gut bacterial structure

**Fig. 4:**
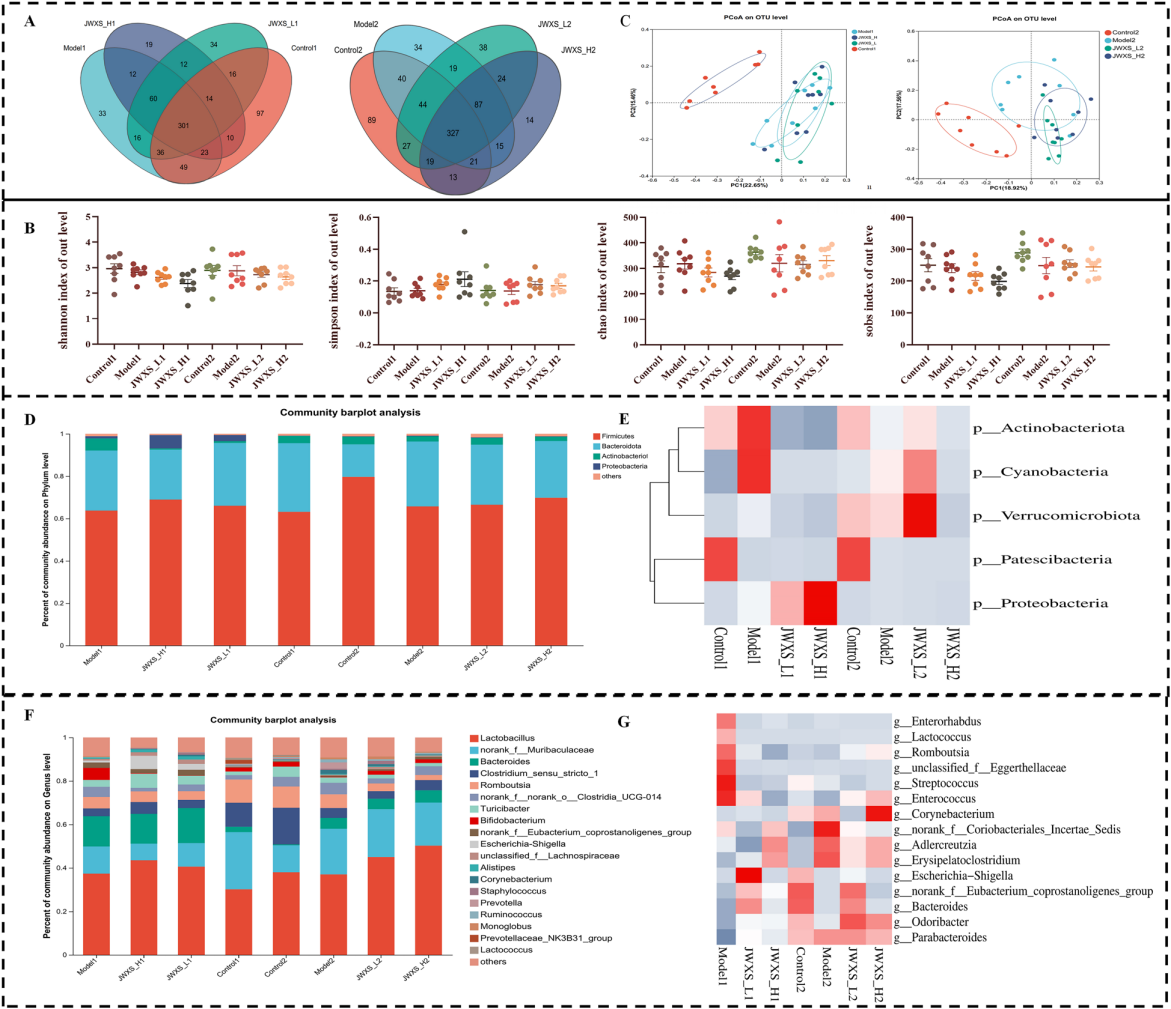
Variation in gut bacteria structure. Data were presented as mean ± sem (n = 8). GraphPad Prism 8.0 was used for statistical analyses. *P < 0.05, **P < 0.01, ***P < 0.001. **A** venn diagrams analysis at 1 week (left) and 2 week (right). **B** Effects of JWXS treatments on Chao, Simpson, Sobs and Shannon diversity indices. **C** PCoA of beta diversity analysis at 1 week (left) and 2 week (right). **D**, **E** Diagram of species structure and differential species at phylum levels in gut microbiota analysis. **F**, **G** Diagram of species structure and differential species at genus levels in gut microbiota analysis

In this study, we assessed the variation at various taxonomic levels. Biological information statistical analysis was conducted on OTUs at a similar level of 97%. The Venn diagram revealed 301 common OTUs at one week and 327 at the end of the modelling period (Fig. 4A). The α diversity was subsequently used to reflect the species richness and evenness. On the basis of the OTU statistical results, the Chao1, Sobs, Ace and Shannon indices were employed to analyze the species change trends. The Sobs and Chao1 indices were used to describe the number of species, whereas the Shannon index was used to depict species diversity. As shown in Fig. 4B, there were no significant differences in the four parameters. Notably, overall species diversity increased with continuous consumption of high-calorie diets. In contrast, the species diversity of the model group displayed a downwards trend in all indicators compared with that of the control group. A recovery in these parameters was observed after JWXS treatment, which aligned with previous findings [4]. In terms of the results, JWXS could increase the richness of the gut microbiota.

With respect to the beta diversity, Bray‒Curtis-based principal coordinate analysis revealed distinct clustering of microbe communities (Fig. 4C). Interestingly, the distance between the JWXS group and the model group increased over time, implying that JWXS treatment revitalized gut microbiota homeostasis. At the phylum level (Fig. 4D), Firmicutes (63.76%), Bacteroidetes (28.41%), Proteobacteria (1.01%), and Actinobacteria (5.70%) accounted for the largest proportions. One-way ANOVA revealed that, compared with those in the control group, the abundances of Actinobacteria, Cyanobacteria, Proteobacteria, and Verrucomicrobiota significantly increased in the model1 group, whereas the abundance of Patescibactera decreased (Fig. 4E, p < 0.05). After two weeks, the overall abundances of Actinobacteria, Cyanobacteria, Verrucomicrobiota, and Patescibacteria in each group increased, but the abundances of Actinobacteria and Cyanobacteria in the model2 group were significantly lower than those in the model1 group. Verrucomicrobiota and Patescibacteria had the opposite results. In addition, the abundance of Proteobacteria in each group significantly decreased. However, the contents in the JWXS treatment group exhibited the opposite trend, and there was no direct correlation with dosage.

Furthermore, the taxonomic genera with significant differences in abundance among the groups at the genus level were analyzed. At the genus level (Fig. 4F), the dominant genera were *Lactobacillus* (37.39%), *Bacteroides* (13.90%), *norank_F_Norank_O_ Clostridia_UCG-014* (12.50%), *Clostridium_Sensu_Stricto_1* (3.57%) and *Romboutsia* (5.29%). Forty-eight differential species were filtered by one-way ANOVA. The heatmap only showed the top 15 differential microbial communities (Fig. 4G). Compared with those in the control1 group, the abundances of *Enterorhabdus*, *Lactococcus*, *Romboutsia*, *Bacteroides* and *Parabacteroides* tended to increase in the model1 group. Instead, the abundances of *Enterorhabdus, Lactococcus, Romboutsia, unclassified_f_Eggerthellaceae*, *Streptococcus* and *Enterococcus* were decreased after 2 weeks. *Corynebacterium*, *norank_f_Coriobacteriales_Incertae_Sedis*, *Adlercreutzia*, *Erysipelatoclostridium*, *Bacteroides*, *Odoribacter*, *Parabacteroides Escherichia-Shigella*, and *norank_f_Eubacterium_coprostanoligenes* were downregulated in the model2 group compared with the control2 group. After the administration of JWXS, the abundance of each genus approached that of the control group, but there was still no correlation with dosage.

In summary, JWXS administration appeared to normalize the abundance levels of each genus, suggesting its potential for ameliorating flora disorders.

### Variation in the gut mycobiome structure

**Fig. 5:**
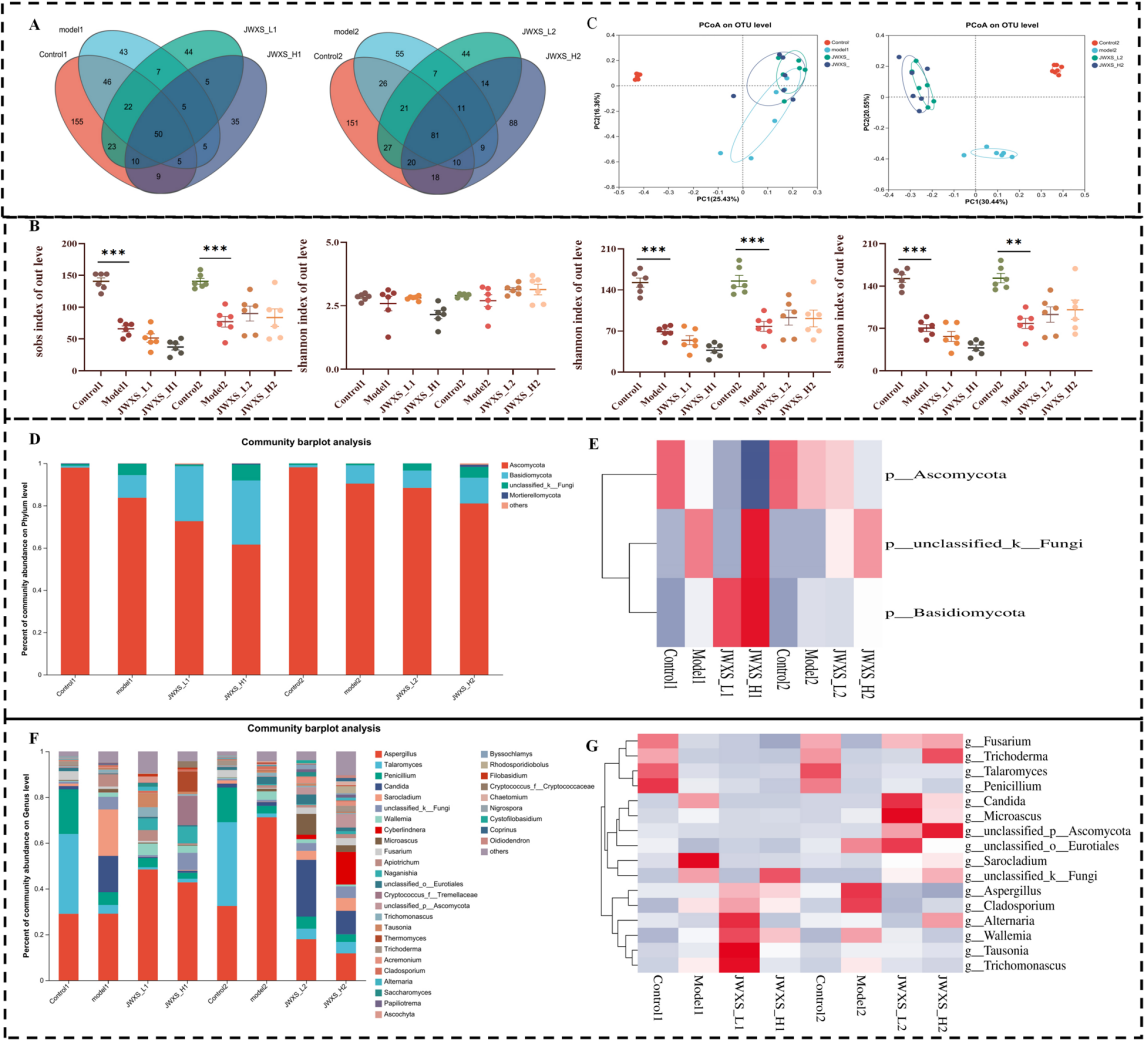
Variation in gut mycobiome structure. Data were presented as mean ± sem (n = 8). GraphPad Prism 8.0 was used for statistical analyses. *P < 0.05, **P < 0.01, ***P < 0.001. **A** venn diagrams analysis at 1 week (left) and 2 week (right). **B** Effects of JWXS treatments on Chao, Simpson, Sobs and Shannon diversity indices. (**C**) PCoA of beta diversity analysis at 1 week (left) and 2 week (right). **D**, **E** Diagram of species structure and differential species at phylum levels in gut microbiota analysis. **F**, **G** Diagram of species structure and differential species at genus levels in gut microbiota analysis

Researchers have proposed that the sources of fungi in the oral cavity and diet could explain all the fungi present in the faeces of healthy subjects, indicating that diet has a significant effect on the composition of intestinal fungi [15]. Hence, the fungal floras were also analyzed. ITS2 analysis revealed changes in the gut mycobiome structure, with 50 common OTUs initially and 81 after two weeks (Fig. 5A). As a result, with the construction of the model, the richness of the species in each group increased. In other words, the microorganism structure changed. As shown in Fig. 5B, no significant changes in the Chao1, Shannon, Sobs and Ace indices were detected. Similar to the variation in bacterial flora, the diversity and abundance of the overall fungal flora increased after two weeks compared with one week. However, compared with the control group, the high-calorie diet of the model2 group significantly reduced fungal diversity. Moreover, the changes in fungal flora were more obvious than those in bacterial flora. Moreover, the PCoA diagram (Fig. 5C) revealed that there was a significant difference among the control2, model2 and JWXS2 groups, with the JWXS dosage showing no correlation with microbial community regulation.

A bar chart was drawn based on the composition and abundance of each group at the phylum and genus levels (Fig. 5D). At the phylum level, the most represented genera were Ascomycota (98%) and Basidiomycota (0.8%). Compared with JWXS administration for 1 week, after two weeks of JWXS administration, the Ascomycota level was downregulated, whereas the Basidiomycota level was upregulated (Fig. 5E). At the genus level (Fig. 5F), the main genera were *Aspergillus, Talaromyces, Candida, Sarocladium* and *Wallemia*. Sixteen differentially abundant genera were identified via one-way ANOVA (Fig. 5G). Compared with those in the control2 group, the richness of *Fusarium*, *Trichoderma*, *Talaromyces*, *Penicillium*, *Microascus*, and *Sarocladium* tended to decrease in the model2 group, whereas the levels of *Aspergillus*, *Candida, Cladosporium*, *Alternaria*, *Wallemia*, *Tausonia* and *Trichomonascus* were increased. As expected, the trend was reversed after JWXS was gavaged, but no correlation with dose was found.

### Differential expression levels of SCFAs in the high-calorie diet

**Fig. 6:**
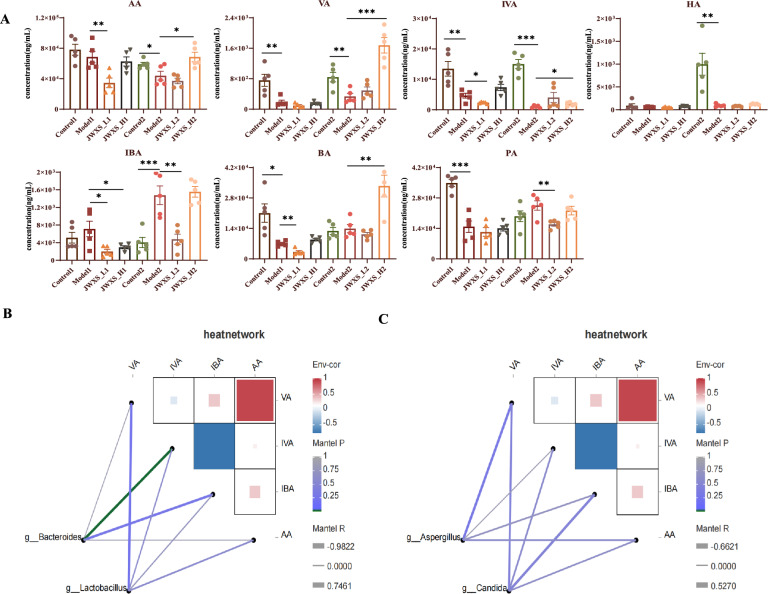
Differential SCFAs identification. Data were presented as mean ± sem (n = 8). GraphPad Prism 8.0 was used for statistical analyses. *P < 0.05, **P < 0.01, ***P < 0.001. (**A**) Seven kinds of SCFAs were detected. **B** Mantel test revealed compositional shifts between bacteria and SCFAs. (**C**) Mantel test revealed compositional shifts between mycobiome and SCFAs

The concentration of SCFAs in the intestine is largely influenced by the composition of the gut microbiota, including the fibre content, digestion time and metabolic flux between the host and microorganisms. SCFAs, crucial bacterial metabolites, play a vital role in human health. In this study, seven major SCFAs were quantified in faecal samples from the control, model and JWXS groups. As shown in Fig. 6A, compared with those in the control2 group, the levels of AA, VA, IVA and HA were significantly lower in the model2 group. In contrast, the levels of IBA, BA and PA increased, although only IBA was significantly different. Following JWXS treatment, the levels of most SCFAs, except for BA, PA, and HA, were adjusted in the opposite direction to the changes observed in the model2 group. These dynamic shifts in SCFAs might be attributable to the continuous accumulation of food. Furthermore, the changes in SCFA content between the JWXS-treated and model2 groups after two weeks were more pronounced than those observed after one week. One week after JWXS administration, some SCFA levels nearly aligned with those of the model1 group. Thus, AA, VA, IVA, and IBA need to be followed with interest.

### Integrated analysis of interactions among genes, fungi, SCFAs and bacteria

The Mantel test was subsequently used to identify associations among bacterial fungi and SCFAs. *Lactobacillus*, *Bacteroides, Aspergillus,* and *Candida* accounted for a large portion of the genera and fungi, respectively. Among them, the relative abundances of these microorganisms were consistently altered during model construction. Compared with those of the model group, the opposite results were observed after JWXS treatment. As shown in Fig. 6B, C, *Lactobacillus* and *Bacteroides* were positively correlated with VA, AA and IVA and were negatively correlated with IBA*.* Surprisingly, completely opposite behaviors were observed for the fungal flora.

A Spearman correlation heatmap displaying the relationships between the DEGs and bacterial flora is shown in Fig. S2A, B. At the genus level, there were negative correlations between the genes (cel, cel2a, cel3b, clps, cpab1, ctrb1, gcat, ggh, loc102554637, pnliprp2, pla2g1b, prss1, reg1a) and *Lactobacillus* and *Bacteroides*. Conversely, *Aspergillus* had a significant positive correlation with these genes.

## Discussion

JWXS, which originated from the Ming Dynasty's Jianpi Pill, is widely recommended for its ease of use and long-term benefits. It is especially beneficial for elderly individuals and children.

JWXS comprises five herbs that maintain the gut. Chemical analysis revealed that *Citrus Reticulatae pericarpium* predominantly contained flavonoids. As reported, pure total flavonoids, including naringin, hesperidin, narirutin, and neohesperidin, can effectively relieve intestinal barrier dysfunction [28]. *Pseudostellariae Radix* consists primarily of cyclic peptides and amino acids, which are known to enhance the absorptive functions of the small intestine. *Crataegi* Fructus is characterized by its alkaloid and organic acid contents, which are associated with digestive aid, lactation support, and anticolitic properties [18]. Polysaccharides are recognized as effective ingredients in *Dioscorea Rhizom*. The ability of Chinese yam polysaccharidein (CYP) to promote gut health is intricately linked to its interactions with gut microbes. In addition, CYPs have potential as anti-inflammatory adjuvants in enteritis therapy [15]. *Hordei Fructus Germinatus* contains both flavonoids and organic acid compounds, with the latter reportedly enhancing gastrointestinal motility in mice and counteracting atropine-induced intestinal smooth muscle relaxation [29].

Bacterial metabolites are important sources of gastrointestinal motility. As reported previously, SCFAs in dietary fibre are fermented by symbiotic bacteria in the gut [30]. In addition to, serving as an important energy source for gastrointestinal motility, they can also directly activate the intestinal nervous system to regulate the synthesis and secretion of certain gastrointestinal hormones by intestinal endocrine cells. The levels of SCFAs were deemed to decrease in functional dyspepsia rats, which aligns with our findings [31].

In this study, AA, VA, IVA and HA were downregulated significantly in the model2 group compared with those in the control2 group, whereas IBA, BA, and PA were upregulated. After JWXS administration, the abundance levels of AA, VA, IVA and IBA approached those of the control2 group, and there was no dose deviation. During model development, the trend for different SCFAs varied slightly, indicating that SCFAs might be predictors of dyspepsia. Numerous experiments on animals have confirmed that SCFAs regulate intestinal motility in relation to their physiological concentrations [32]. Intestinal motility was enhanced at SCFA concentrations of 10–100 mmol/L but inhibited when concentrations exceeded 100 mmol [33]. Therefore, the concentration of SCFAs directly affects colonic motility. In this study, we observed that JWXS could significantly modulate alterations in AA, VA, IVA, and IBA. VA, BA, and IVA are associated primarily with lipid metabolism and inflammation. Given that the gastrointestinal tract is notably impacted by indigestion, the vesicular protrusions observed on the stomach surface in our study might be linked to inflammation and dynamic changes in SCFAs. AA, constituting a major portion of SCFAs in the intestine, is pivotal in regulating internal pH and maintaining environmental stability. AA is produced by various bacteria, including *Lactobacillus*. Through Pearson correlation analysis, *Lactobacillus* and *Bacteroides* were found to be positively correlated with VA, AA, and IVA. Studies have indicated that AA can effectively maintain mucosal integrity, reduce intestinal barrier permeability, and decrease the penetration of harmful substances by increasing the expression of tight junction proteins in host intestinal epithelial cells, thereby improving indigestion [34]. Additionally, AA can regulate the composition of the gut microbiota, increase the number of beneficial bacteria, reduce the number of pathogenic bacteria, increase the number of SCFA-producing bacteria, and increase faecal and serum SCFA levels [35]. This finding aligns with those of with our study, which revealed a significant decrease in the levels of VA, AA, and IVA in the faecal samples of the model group through targeted metabolomics. After JWXS administration, VA, AA, and IVA all exhibited a certain degree of recovery. Similarly, the contents of the major genera *Lactobacillus* and *Bacteroides* also increased following administration. *Lactobacillus* and *Bacteroides* were reported as beneficial bacteria, indeed played a positive role in improving conditions of indigestion. For instance, *Lactobacillus* could break down cellulose and other indigestible substances in food, promoting digestion and nutrient absorption [35]. *Bacteroides* played a crucial role in regulating the composition of the gut microbiota and promoting the production of short-chain fatty acids (SCFAs) in the intestine [36]. In addition to bacteria and viruses, the human gastrointestinal tract hosts a significant fungal population, these form a complex biological network which must maintain a balanced state within the body. [37]. A crucial homeostatic mechanism arises from microbial competition, which keeps the relative abundance of microbial species in a healthy balance. A decrease in bacterial abundance was followed by an increase in fungal abundance in the model group, demonstrating a certain competitive relationship between them. Fungi might indirectly affect the production of SCFAs by influencing the activity of these bacteria [38], and SCFAs might also serve as potential regulatory factors in the symbiotic and pathogenic mechanisms of fungi [39]. Fungal growth and reproduction within the host can cause intestinal damage and potentially trigger inflammatory bowel disease. In our study, differential and Pearson correlation analyses revealed that the levels of *Aspergillus* and *Candida* significantly increased in the model group. The increase in *Aspergillus and Candida* bacteria might invade intestinal epithelial cells, resulting in impaired intestinal barrier function, increased intestinal permeability, and ultimately indigestion. JWXS administration significantly reduced the levels of these fungal species [40]. Changes in the gut microbiome were a common manifestation of pancreatic damage and characterized by a reduction in Firmicutes and Actinobacteria and an increase in the Bacteroidetes phylum. The pancreas may be affected not only by direct colonization by bacteria but also by small molecules arising from dysbiosis of the gut microbiota [41, 42]. The pancreas releases proteases (such as PRSS, CTRB1, CELA, CPA, and CPB) into the small intestine to further digest proteins into smaller peptides and amino acids. Undigested proteins reach the distal colon, where colonic bacteria ferment them. This process generates short-chain fatty acids (SCFAs), which are important for colon health. This study also revealed that dietary proteins, particularly elastin and collagen, can be metabolized by both bacterial and fungal enzymes in the small intestine, highlighting the complex interactions between dietary components and the microbiome.

Animal and clinical studies have elucidated the influence of the gut microbiota on several pathogenic mechanisms, including impaired gastrointestinal motility, visceral sensitivity, immune activation, increased mucosal permeability and alterations in the gut‒brain axis [7]. Spearman correlation revealed the relationships between the DEGs and the bacterial flora. Notably, *Lactobacillus* and *Bacteroides* presented significant negative correlations among genes (cel, cel2a, cel3b, clps, cpab1, ctrb1, gcat, ggh, loc102554637, pnliprp2, pla2g1b, prss1, and reg1a). Similarly, a significant postive correlation was observed with the fungal community. Therefore, these key DEGs warranted further attention in our research. Further enrichment analysis focused on the pancreatic secretion pathway. Previous research has indicated that genes such as cel3b, cpa1, ctrb1, prss1, clps, cel2a, cpb1, and try5 are secreted into the pancreas as proenzymes or cofactors. For example, prss1 encodes a pancreatic proenzyme, a member of the serine protease family [43], which is secreted by the pancreas and activated in the small intestine. Clps, cofactors essential for the efficient hydrolysis of dietary fats by pancreatic lipase, are exclusively expressed in pancreatic acinar cells, suggesting the presence of tissue-specific regulatory elements. These genes are associated with the risk of pancreatitis.

Cel3b, prss1, and clps primarily aid in fat digestion, promoting fat absorption. On the other hand, cpa1, cpb1, try5, cel2a, and ctrb1 are involved in the breakdown of dietary proteins to facilitate protein digestion. WB-based quantitative validation of Cpa1, cel2a, and ctrb1 revealed that the demand for digestive enzymes increased with a high-protein diet. Meanwhile, *Lactobacillus* and *Bacteroides* decreased and *Aspergillus* increased in the model group. This implied that indigestion caused by high-protein diets might be reflected at the genetic level as an increased demand for digestive enzymes, and at the microbiota level as a reduction in the content and types of beneficial bacteria, along with an increase in pathogenic fungi. JWXS might contribute to alleviating conditions of indigestion and maintaining stable pancreatic secretion. Pancreatic enzyme secretion is a crucial factor influencing poor digestion in the body [44].

**Fig. 7:**
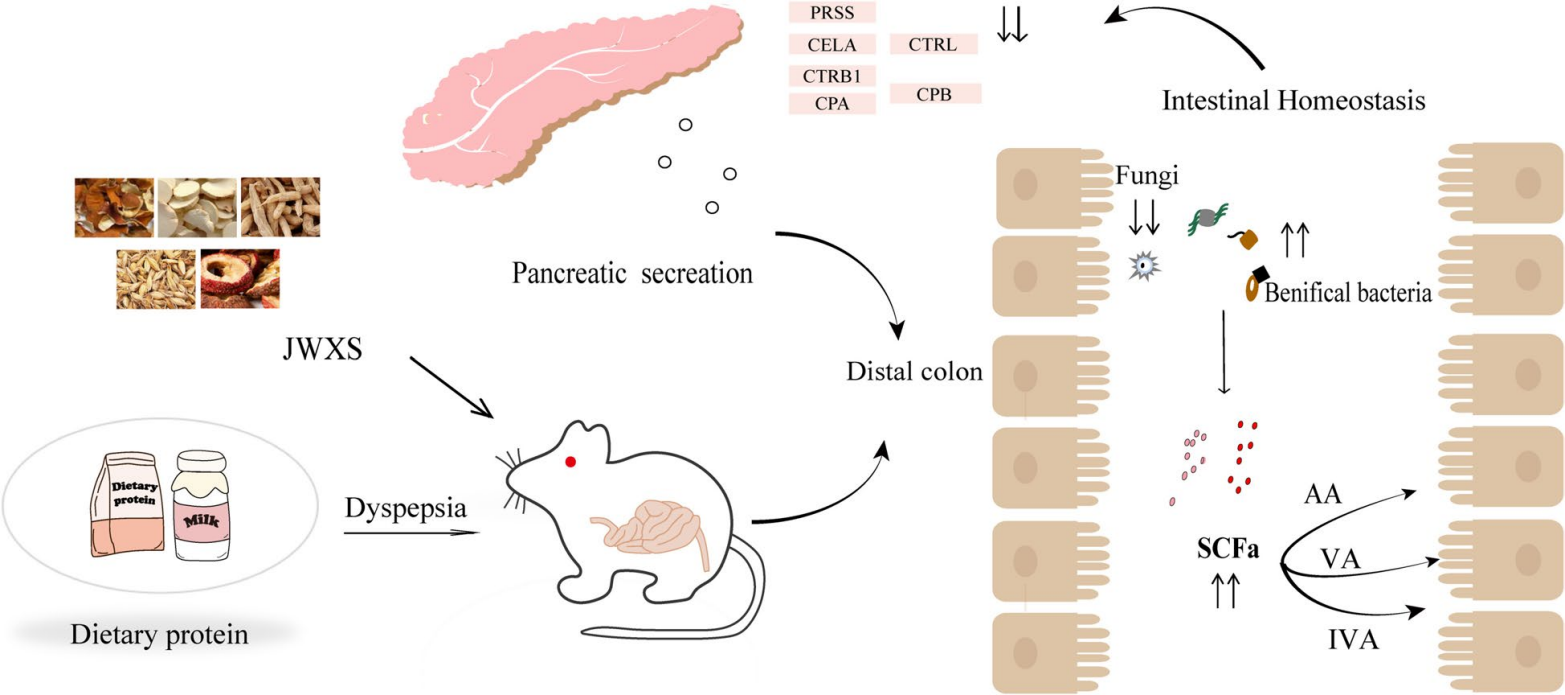
The dyspepsia induced dysbiosis of gut microbiota, metabolites and genes leading to significant differences in the predicted functional profiles

The in vivo action process corresponding to these interactions is depicted in Fig. 7. The overall process highlights the importance of protein digestion for nutrient absorption and the role of the gut microbiota in fermenting undigested proteins, contributing to the gut environment and potentially to overall health.

Indeed, we acknowledged that there were limitations to our study. The identification of active components was primarily conducted through network pharmacology screening, and a more in-depth investigation of these components was warranted. Furthermore, the competitive relationship between bacteria and fungi had yet to be sufficiently validated through experimentation. Therefore, we proposed that future studies incorporate in *vivo* activity assays and co-cultivation techniques to enhance the inference of causal relationships.

## Conclusion

In our research, we tentatively or unambiguously identified a total of 105 chemical compounds, including 56 flavonoids, 11 alkaloids, 1 triterpenoid, 11 organic acids, 10 cyclic peptides, 3 amino acids, 2 sugars, and 11 compounds from other classes. This identification was achieved through comparisons with standard substances and literature references. Additionally, we successfully established a dyspepsia model in rats via a high-calorie diet. JWXS was proven to improve gastrointestinal function damage in immature rats fed a high-calorie diet via polypharmacology verification. Our transcriptomic analysis and gut microbiota interaction studies provide compelling evidence that JWXS exerts its digestive effects primarily through the pancreatic secretion signalling pathway, involving key genes such as try5, cel2a, ctrb1, cel3b, cpa1, cpb1, prss1 and clps. Furthermore, the critical genes were validated by RT-qPCR and WB experiments.

Importantly, our study represented the first investigation into continuous-state microbiota changes during indigestion. We observed significant microbiota (bacteria and fungi) alterations mediated by SCFAs such as AA, IVA, and VA. An analysis of the main genera of bacteria and fungi revealed that *Lactobacillus* and *Bacteroides* were significantly reduced in the model group, whereas *Aspergillus* and *Candida* were significantly increased. Further validation via metabolomics revealed that AA, IVA, and VA were significantly reduced in the model group, with positive correlations with *Lactobacillus* and *Bacteroides* and negative correlations with *Aspergillus* and *Candida*. These findings suggest that JWXS might achieve the goal of treating indigestion by alleviating microbial disturbances.

These findings lay a theoretical foundation for understanding the digestion mechanism of JWXS in immature rats. This knowledge not only expands the potential applications of JWXS but also provides a valuable reference for its rational use in treating dyspepsia.

## Supplementary Information

Additional file 1.

Additional file 2.

Additional file 3.

## Data Availability

The RNA-seq data of duodenum presented in the study are deposited in the GEO data, accession number is GSE245835. The fungal and bacterial sequencing data can be obtained from BioProject PRJNAPRJNA1030804 and PRJNA1030796.

## Abbreviations

AA: Acetic acid
BA: Butyric acid
Clps: Colipase
CYP: Chinese yam polysaccharidein
Ctrb1: Chymotrypsin B1
Cel2a: Chymotrypsin-like elastase family member 2A
Cel3b: Chymotrypsin-like elastase family member 3B
Cpa1: Carboxypeptidase A1
Cpb1: Carboxypeptidase B1
Prss1: Serine Protease 1
DEGs: Differential expressed genes
GB: Giga base
HA: Hexanoic acid
IVA: Isovaleric acid
IBA: Isobutyric acid
4-MA: 4-Methylvaleric acid
JWXS: Jianwei Xiaoshi Oral Liquid
KEGG: Kyoto Encyclopedia of Genes and Genomes
GO: Gene ontology
PA: Propionic acid
PCA: Principal component analysis
Prss1: Serine Protease 1
RT-qPCR: Real-time quantitative PCR
SD: Sprague Dawley
SEM: Standard error of the mean
TCM: Traditional Chinese medicine
Try5: Trypsin-5
VA: Valeric acid
