# Supplementary material for: Jianwei Xiaoshi oral liquid attenuates high-calorie diet-induced dyspepsia in immature rats via regulating the pancreatic secretion pathway and maintaining the homeostasis of intestinal microbiota
Source: Chin Med. 2025 Jan 4;20:6. doi: 10.1186/s13020-024-01052-3 (PMC11700448; doi:10.1186/s13020-024-01052-3)
Supplement: Supplementary file 1 — Additional file 1. [file 13020_2024_1052_MOESM1_ESM.docx]

**Table S1 Primer sequences of 8 differentially expressed genes**

| gene symbol | Primer | 5' to 3' |
| --- | --- | --- |
| Cela2a | Forward primer | GCATGATGTGAGCAGGGTA |
|  | Reverse primer | CTGAACAGCCAGTGAGCC |
| Cela3b | Forward primer | GACGCCGTTCCCTACAGT |
|  | Reverse primer | GGTGCGAGAAGTCGAGATG |
| Cpa1 | Forward primer | CCCCATTGATGTCAGAGTG |
|  | Reverse primer | ATCCAGCGTATGATAGGTG |
| Cpb1 | Forward primer | CTTGTCACTCAGAGCGTCAT |
|  | Reverse primer | CCACAGGCAGAACATAGAA |
| Ctrb1 | Forward primer | GCTCCTGTCCTGCTTTGC |
|  | Reverse primer | ACCCAGTCCTCGCTGATG |
| Prss1 | Forward primer | CCCTTTGGAAGATGATGAC |
|  | Reverse primer | TTGGATGCGGGATTTGTA |
| Try5 | Forward primer | GAGGGCAATGAGCAGTTT |
|  | Reverse primer | GAGCAGGTCTGGGTTGTT |
| Clps | Forward primer | GCTCTGCTTGTAACCCTCG |
|  | Reverse primer | GCACTCGCTGTTCTCCAT |
| β-actin | Forward primer | TTCGCCATGGATGACGATATC |
| internal reference | Reverse primer | TAGGAGTCCTTCTGACCCATAC |

**Table S2 ADME parameters information of each compound**

| Molecule | Formula | MW | GI absorption | Lipinski #violations | Ghose #violations | Veber #violations | Egan #violations | Muegge #violations | Bioavailability Score | source |
| --- | --- | --- | --- | --- | --- | --- | --- | --- | --- | --- |
| Gramine | C_11_H_14_N_2_ | 174.24 | High | Yes | Yes | Yes | Yes | No | 0.55 | A |
| Synephrine | C_9_H_13_NO_2_ | 167.21 | High | Yes | Yes | Yes | Yes | No | 0.55 | C |
| L-Leucine | C_6_H_13_NO_2_ | 131.17 | High | Yes | No | Yes | Yes | No | 0.55 | B |
| 1,7-Bis(4-hydroxyphenyl)-3,5-heptanediol | C_19_H_24_O_4_ | 316.39 | High | Yes | Yes | Yes | Yes | Yes | 0.55 | C |
| N-methyltyramine | C_9_H_13_NO | 151.21 | High | Yes | No | Yes | Yes | No | 0.55 | A |
| Hordenine | C_10_H_15_NO | 165.23 | High | Yes | Yes | Yes | Yes | No | 0.55 | A |
| Phloroglucinol | C_6_H_6_O_3_ | 126.11 | High | Yes | No | Yes | Yes | No | 0.55 | D |
| Phenylalanine | C_9_H_11_NO_2_ | 165.19 | High | Yes | Yes | Yes | Yes | No | 0.55 | A,B |
| D-Pantothenic Acid | C_9_H_17_NO_5_ | 219.23 | High | Yes | No | Yes | Yes | Yes | 0.56 | B |
| Vanillic acid | C_8_H_8_O_4_ | 168.15 | High | Yes | Yes | Yes | Yes | No | 0.85 | B |
| Coumaroyl-hydroxyagmatine | C_14_H_20_N_4_O_3_ | 292.33 | High | Yes | Yes | Yes | Yes | Yes | 0.55 | A |
| Feruloyl putrescine | C_14_H_20_N_2_O_3_ | 264.32 | High | Yes | Yes | Yes | Yes | Yes | 0.55 | A |
| p-Coumaroylagmatine | C_14_H_20_N_4_O_2_ | 276.33 | High | Yes | Yes | Yes | Yes | Yes | 0.55 | A |
| Sinapoyl agmatine | C_16_H_24_N_4_O_4_ | 336.39 | High | Yes | Yes | Yes | No | Yes | 0.55 | A |
| Eucomic acid | C_11_H_12_O_6_ | 240.21 | High | Yes | Yes | Yes | Yes | Yes | 0.56 | B |
| Caffeic acid | C_9_H_8_O_4_ | 180.16 | High | Yes | Yes | Yes | Yes | No | 0.56 | D |
| Quercetin | C_15_H_10_O_7_ | 302.24 | High | Yes | Yes | Yes | Yes | Yes | 0.55 | B,D,E |
| Diosmetin | C_16_H_12_O_6_ | 300.26 | High | Yes | Yes | Yes | Yes | Yes | 0.55 | C |
| Hannokinol | C_19_H_24_O_4_ | 316.39 | High | Yes | Yes | Yes | Yes | Yes | 0.55 | B |
| 9,10-dihydroxy-8-oxo-12-octadEcenoic acid | C_18_H_32_O_5_ | 328.44 | High | Yes | Yes | No | Yes | Yes | 0.56 | A |
| Pinellic Acid | C_18_H_34_O_5_ | 330.46 | High | Yes | Yes | No | Yes | Yes | 0.56 | A |
| Hesperetin | C_16_H_14_O_6_ | 302.28 | High | Yes | Yes | Yes | Yes | Yes | 0.55 | C |
| Isosinensetin | C_20_H_20_O_7_ | 372.37 | High | Yes | Yes | Yes | Yes | Yes | 0.55 | C |
| 5,6,7,3,4',5'-hexamethoxyflavone | C_21_H_22_O_8_ | 402.39 | High | Yes | Yes | Yes | Yes | Yes | 0.55 | C |
| Tetramethoxyflavone | C_19_H_18_O_6_ | 342.34 | High | Yes | Yes | Yes | Yes | Yes | 0.55 | C |
| 5，6，7，4'-tetramethoxyflavone | C_26_H_24_O_7_ | 448.46 | High | Yes | Yes | Yes | Yes | Yes | 0.55 | C |
| Batatasin III | C_15_H_16_O_3_ | 244.29 | High | Yes | Yes | Yes | Yes | Yes | 0.55 | B |
| Limonin | C_26_H_30_O_8_ | 470.51 | High | Yes | Yes | Yes | Yes | Yes | 0.55 | C |
| 5,7,8,3,4',5'-hexamethoxyflavone | C_21_H_22_O_8_ | 402.39 | High | Yes | Yes | Yes | Yes | Yes | 0.55 | C |
| 5,7, 4'-trimethoxyflavone | C_18_H_16_O_6_ | 328.32 | High | Yes | Yes | Yes | Yes | Yes | 0.55 | C |
| Sinensetin | C_20_H_20_O_7_ | 372.37 | High | Yes | Yes | Yes | Yes | Yes | 0.55 | C |
| Nobiletin | C_21_H_22_O_8_ | 402.39 | High | Yes | Yes | Yes | Yes | Yes | 0.55 | C |
| Tetramethoxyflavone isomer | C_19_H_18_O_6_ | 342.34 | High | Yes | Yes | Yes | Yes | Yes | 0.55 | C |
| Liquiritigenin | C_15_H_12_O_4_ | 256.25 | High | Yes | Yes | Yes | Yes | Yes | 0.55 | B |
| 9,10-dihydroxy-12Z-octadecenoic acid | C_18_H_34_O_4_ | 314.46 | High | Yes | Yes | No | Yes | Yes | 0.56 | A |
| Gardenin A | C_21_H_22_O_9_ | 418.39 | High | Yes | Yes | Yes | Yes | Yes | 0.55 | C |
| Maslinic acid | C_30_H_48_O_4_ | 472.7 | High | Yes | No | Yes | No | No | 0.56 | B |
| Vanillin | C_8_H_8_O_3_ | 152.15 | High | Yes | No | Yes | Yes | No | 0.55 | D |
| Acacetin | C_16_H_12_O_5_ | 284.26 | High | Yes | Yes | Yes | Yes | Yes | 0.55 | D |
| Batatasin I | C_17_H_16_O_4_ | 284.31 | High | Yes | Yes | Yes | Yes | Yes | 0.55 | B |
| Catechin | C_15_H_14_O_6_ | 290.27 | High | Yes | Yes | Yes | Yes | Yes | 0.55 | B |
| Epicatechin | C_15_H_14_O_6_ | 290.27 | High | Yes | Yes | Yes | Yes | Yes | 0.55 | B |
| Syringic acid | C_9_H_10_O_5_ | 198.17 | High | Yes | Yes | Yes | Yes | No | 0.56 | B |

^A^ *Hordei Fructus* Germinatus.; ^B^ *Dioscoreae Rhizoma*; ^C^ *Citrus Reticulatae Pericarpium;* ^D^ *Crataegi* Fructus; ^E^ *Pseudostellariae Radix*.
