# Supplementary figures and images for: Jianwei Xiaoshi oral liquid attenuates high-calorie diet-induced dyspepsia in immature rats via regulating the pancreatic secretion pathway and maintaining the homeostasis of intestinal microbiota
Source: Chin Med. 2025 Jan 4;20:6. doi: 10.1186/s13020-024-01052-3 (PMC11700448; doi:10.1186/s13020-024-01052-3)

A

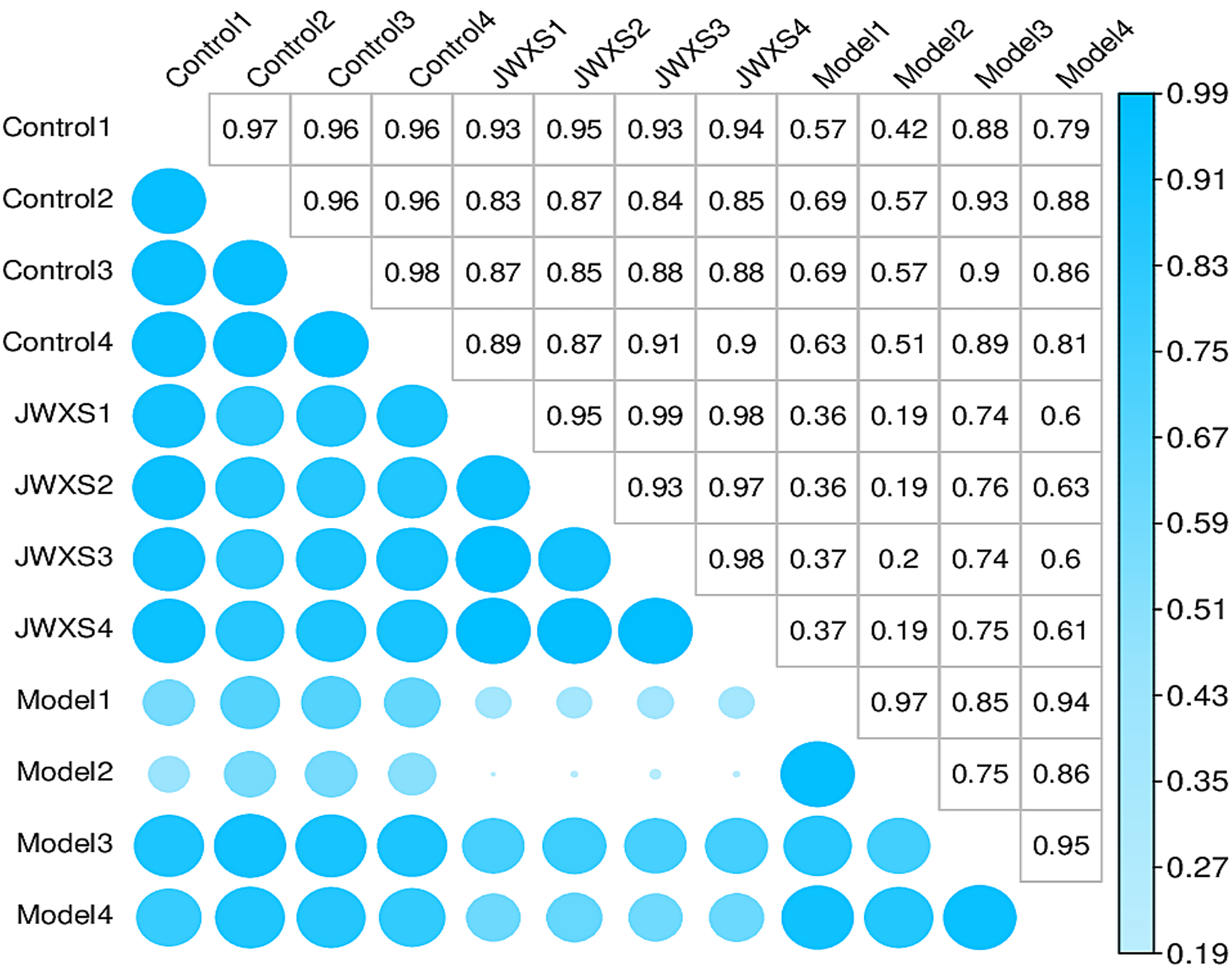

B

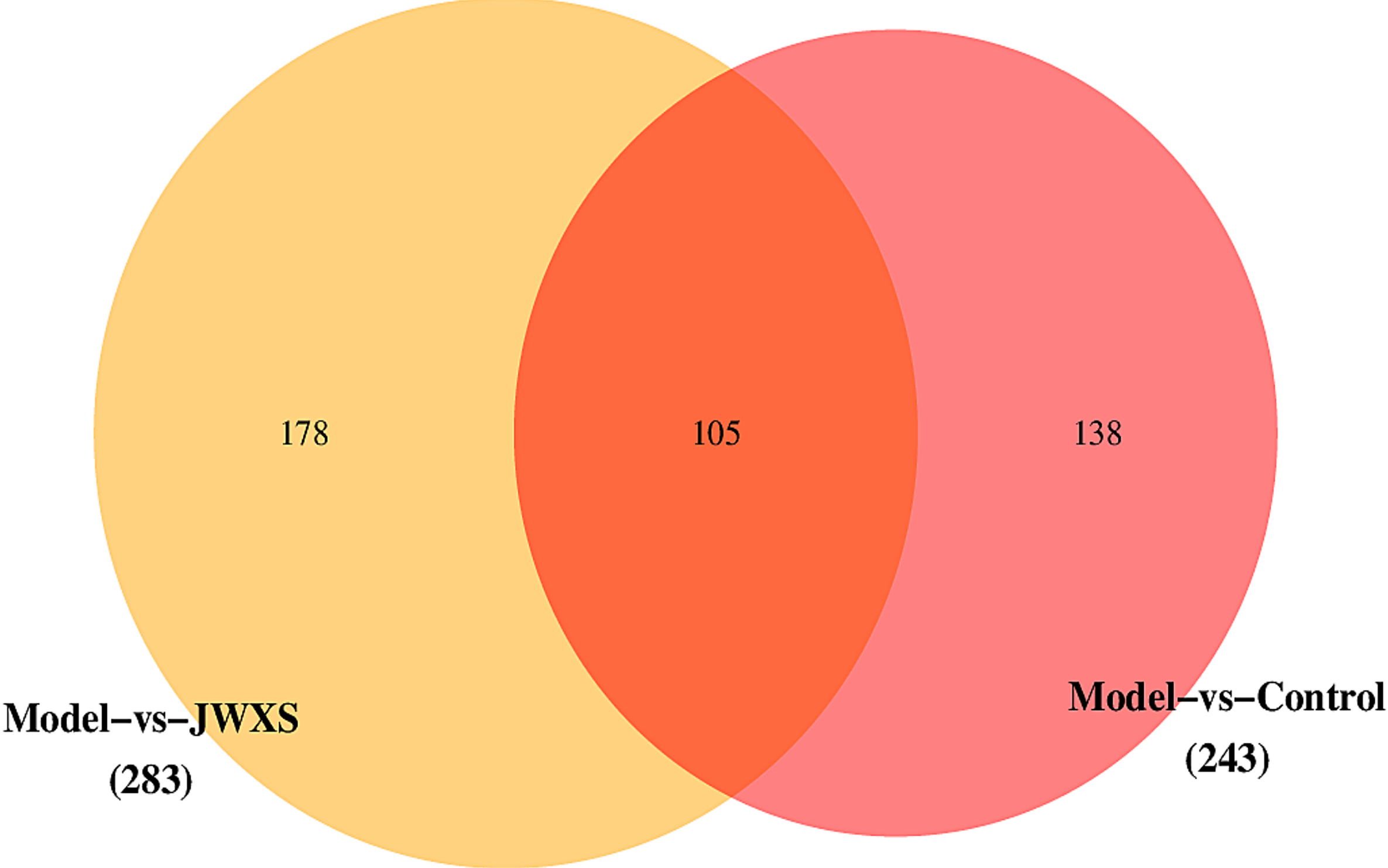

C

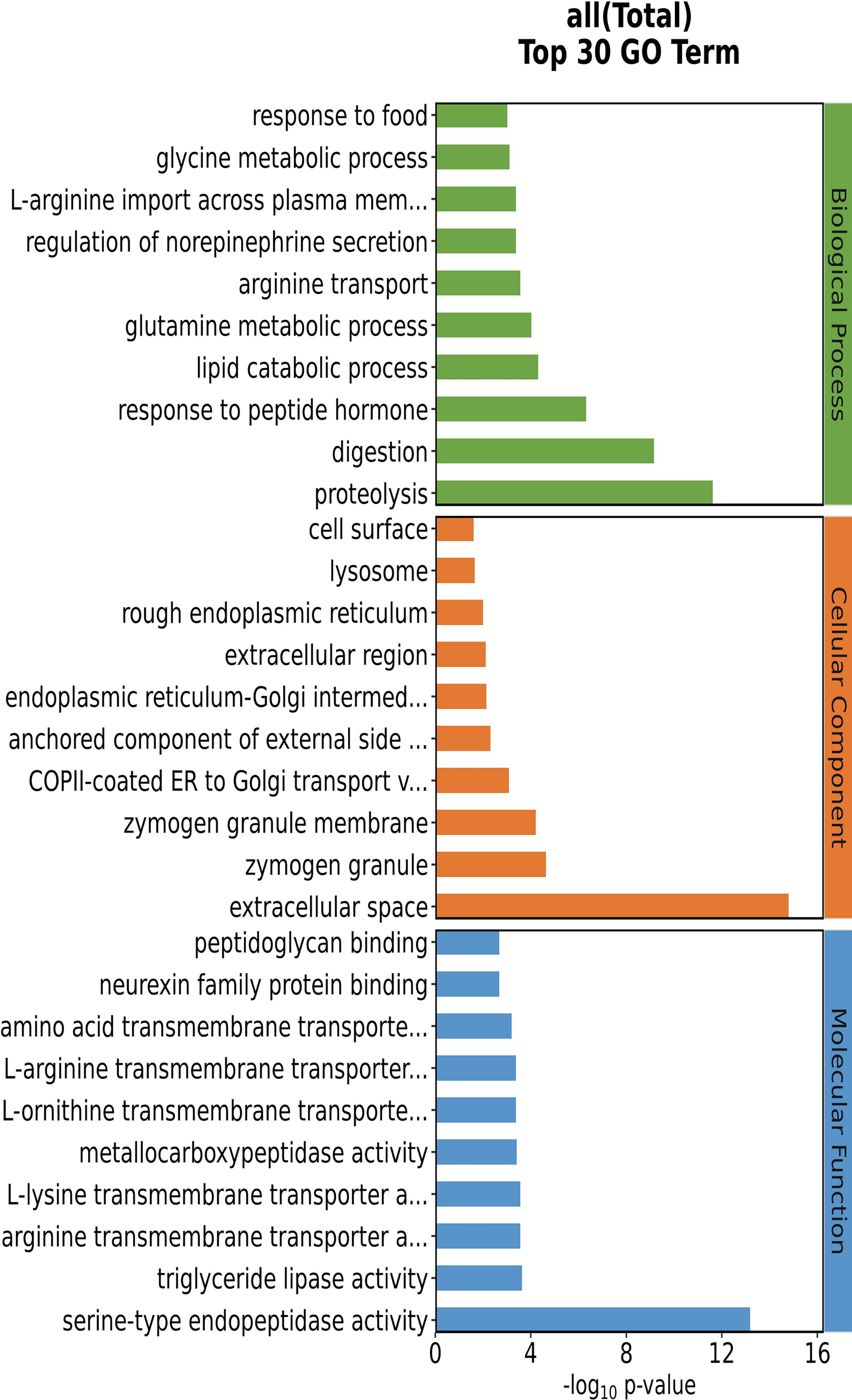

Supplement: Supplementary file 2 — Additional file 2. [file 13020_2024_1052_MOESM2_ESM.pdf]

Spearman Correlation Heatmap

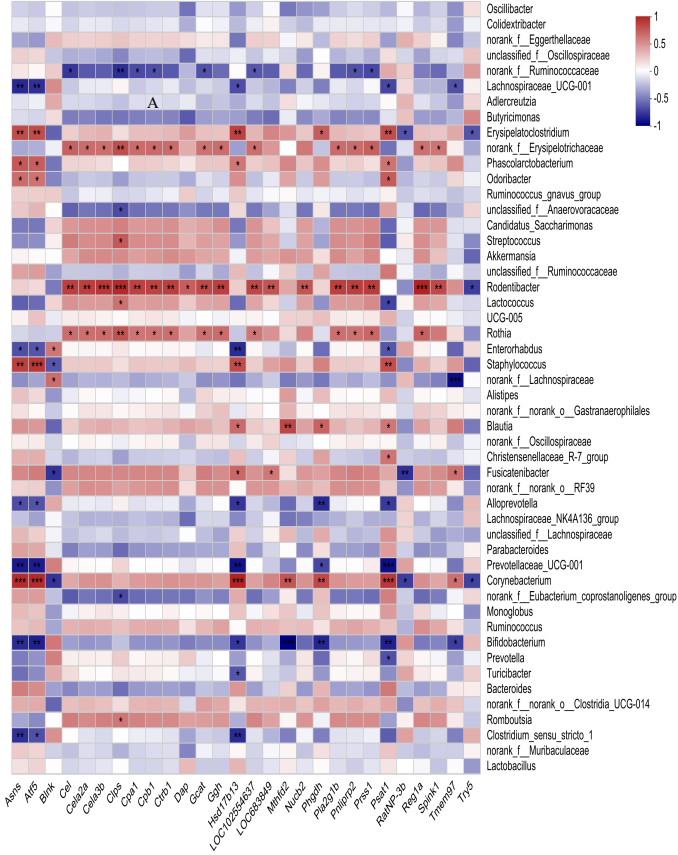

B

Spearman Correlation Heatmap

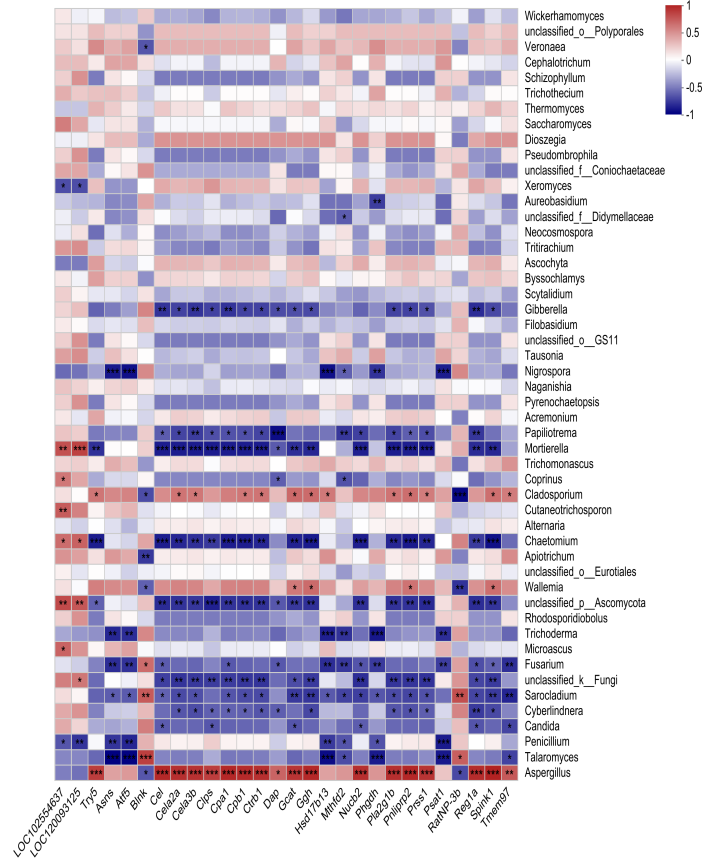

Supplement: Supplementary file 3 — Additional file 3. [file 13020_2024_1052_MOESM3_ESM.pdf]
